# Combinatorial Detection of Conserved Alteration Patterns for Identifying Cancer Subnetworks

**DOI:** 10.1093/gigascience/giz024

**Published:** 2019-04-11

**Authors:** Ermin Hodzic, Raunak Shrestha, Kaiyuan Zhu, Kuoyuan Cheng, Colin C Collins, S Cenk Sahinalp

**Affiliations:** 1 Laboratory for Advanced Genome Analysis, Vancouver Prostate Centre, 2660 Oak St, Vancouver, BC, V6H 3Z6, Canada; 2 School of Computing Science, Simon Fraser University, 8888 University Dr, Burnaby, BC, V5A 1S6, Canada; 3 Department of Urologic Sciences, University of British Columbia, 2775 Laurel St, Vancouver, BC, V5Z 1M9, Canada; 4 Department of Computer Science, Indiana University Bloomington, 700 N. Woodlawn Ave, Bloomington, IN, 47408, USA; 5 Center for Bioinformatics and Computational Biology, University of Maryland, 8125 Paint Branch Dr, College Park, MD, 20742, USA

**Keywords:** alterations, cancer, combinatorial optimization, conserved subnetwork

## Abstract

**Background:**

Advances in large-scale tumor sequencing have led to an understanding that there are combinations of genomic and transcriptomic alterations specific to tumor types, shared across many patients. Unfortunately, computational identification of functionally meaningful and recurrent alteration patterns within gene/protein interaction networks has proven to be challenging.

**Findings:**

We introduce a novel combinatorial method, cd-CAP (**c**ombinatorial **d**etection of **c**onserved **a**lteration **p**atterns), for simultaneous detection of connected subnetworks of an interaction network where genes exhibit conserved alteration patterns across tumor samples. Our method differentiates distinct alteration types associated with each gene (rather than relying on binary information of a gene being altered or not) and simultaneously detects multiple alteration profile conserved subnetworks.

**Conclusions:**

In a number of The Cancer Genome Atlas datasets, cd-CAP identified large biologically significant subnetworks with conserved alteration patterns, shared across many tumor samples.

## Introduction

Recent large-scale tumor-sequencing projects such as Pan Cancer Analysis of Whole Genomes (PCAWG) have revealed a multitude of somatic genomic, transcriptomic, proteomic, and epigenomic alterations across cancer types. However, only a select few of these alterations provide proliferative advantage to the tumor and hence are called "driver" alterations [1]. Distinguishing driver alterations from functionally inconsequential random "passenger" alterations is critical for therapeutic development and cancer treatment.

Cancers are often driven by alterations to multiple genes [2,3]. Whereas genomic alterations are likely consequences of endogenous or exogenous mutagen exposures [4], their evolutionary selection depends on the functional role of the affected genes [1] and their synergistic combinations. For example, *TMPRSS2*-*ERG* gene fusion is an early driver event in almost half of prostate cancer cases, and it often co-exists with copy number loss of *PTEN* and *NKX3-1* [5–7]. Another example is the concomitant deletion of 4 cancer genes*—BAP1, SETD2, PBRM1*, and *SMARCC1—*in chromosome locus 3p21, identified as a driver event in clear cell renal cell carcinoma [8], uveal melanoma [9], and mesotheliomas [10]. These genes are involved in the chromatin remodeling process, and their loss further impairs the DNA damage repair pathway in tumors [9].

Alterations in ≥2 genes might be evolutionary co-selected because alteration in 1 gene might enhance the deleterious effect of the others [11]. Such co-selected genes are often active in a functionally significant subnetwork (i.e., module or pathway) within the human gene/protein interaction network, and aberrations in such subnetworks are common to particular cancer types as demonstrated by recent sequencing efforts (e.g., PCAWG) [12]. For instance, *TMPRSS2* interacts with *ERG* and *PTEN* (see the example above) in the STRING version 10 protein-protein interaction (PPI) network; in fact all 3 genes co-operate to modulate the NOTCH signaling pathway in *TMPRSS2*-*ERG*–positive prostate cancer progression [7]. As a result, it is desirable to identify subsets of functionally interacting genes that are commonly (genomically or transcriptomically) altered in specific tumor types.

Recently, a number of computational methods have been developed to identify recurrent genomic (as well as transcriptomic) alteration patterns across tumor samples. Some of these methods have been designed to identify multiple gene alterations simultaneously on the basis of their co-occurrence or mutual exclusivity relationships in a tumor cohort, either with [13] or without [14,15] reference to a molecular interaction network. Other methods have been developed to identify subnetworks within a molecular interaction network with specific characteristics, e.g., the subnetwork of a fixed size with the highest total "weight" [16,17] or the subnetwork seeded by a particular node that can be derived through a diffusion process [18,19]; naturally these methods do not capture recurrent alteration patterns across a cohort. A direction particularly relevant to our article is motivated by a number of related works [18,20–22] and explored by Bomersbach et al. [23], which suggests finding a subnetwork of a given size *k* with the goal of maximizing *h*, the number of samples for which ≥1 gene of the subnetwork is in an altered state. (A similar formulation where the goal is to maximize a weighted difference of *h* and *k*, for varying size *k*, can be found in Hristov and Singh [24].) Although the above combinatorial problems are typically NP-hard, they became manageable through the use of state of the art integer linear programming (ILP) solvers or greedy heuristics, or by the use of complex preprocessing procedures that substantially reduce the problem size.

Complementary to the ideas proposed above, there are also several approaches to identify mutually exclusive (rather than jointly altered) sets of genes and pathways [25–27]. These approaches utilize the mutational heterogeneity prevalent in cancer genomes and are driven by the observation that mutations acting on same pathway are often mutually exclusive across tumor samples. Although, from a methodological point of view, these approaches are very interesting, they are not trivially extendable to the problem of identifying co-occurring alteration patterns (involving >2 genes) conserved across many samples.

### Our contribution

**Figure 1:**
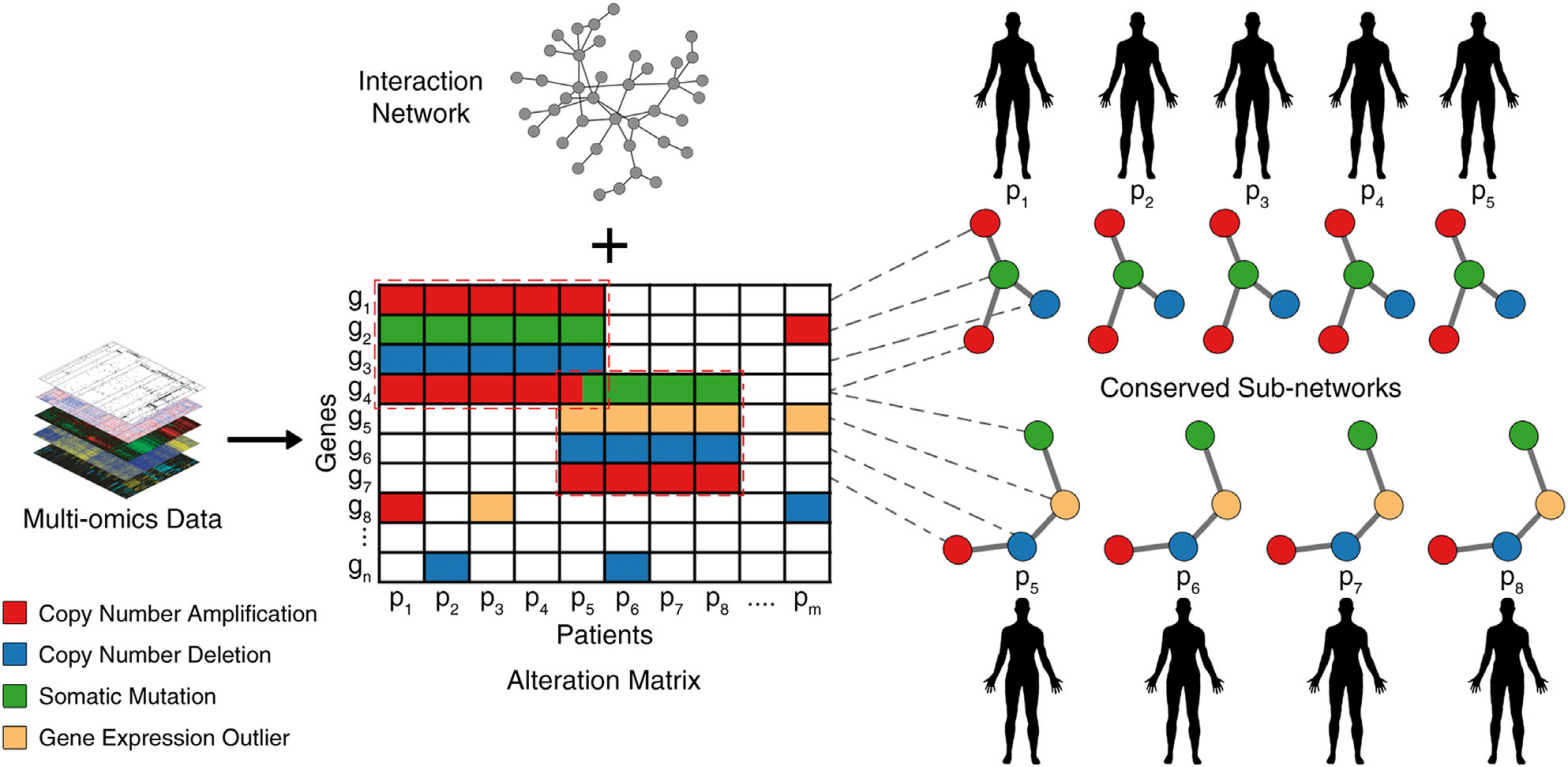
Schematic Overview of our framework. Multi-omics alteration profiles of a cohort of tumor samples are identified using appropriate bioinformatics tools. The alteration information is combined with gene-level information in the form of a sample-gene alteration matrix. Each alteration type is assigned a distinct color. Using a (signaling) interaction network, cd-CAP identifies subnetworks with conserved alteration patterns.

In this article we present a novel computational method, **c**ombinatorial **d**etection of **c**onserved **a**lteration **p**atterns (cd-CAP), for detection of subnetworks of an interaction network, each with an alteration pattern conserved across a large subset of a tumor sample cohort. The framework of cd-CAP allows each gene to be labeled (or "colored") with ≥1 distinct alteration type (e.g., somatic mutation, copy number alteration, or aberrant expression) with the goal of identifying ≥1 subnetworks, each with a specific alteration (labeling) pattern, that is shared across many samples (Fig. 1). As such, cd-CAP solves a novel problem that has not been tackled in the literature. In fact, the very notion of "conserved subnetworks" used by cd-CAP is novel: in Bomersbach et al. [23] and Hristov and Singh [24] the subnetworks of interest are composed of nodes such that in each patient ≥1 is altered (one way or another). In contrast, cd-CAP insists that each node is altered in each patient, and each node preserves its alteration type in each patient. Additionally, unlike Hristov and Singh [24], which employ heuristics to solve a highly restrictive problem and thus cannot guarantee optimality, cd-CAP uses a very efficient exhaustive search method (a variant of the a priori algorithm, originally designed for association rule mining [28]) to quickly solve a very general problem optimally.

cd-CAP offers 2 basic modes: the "single-subnetwork" mode identifies the largest subnetwork altered the same way in ≥*t* samples by solving the maximum conserved subnetwork identification problem (MCSI) optimally; the "multi-subnetwork" mode identifies *l* subnetworks of size (at most) *k* (*k* and *l* are user-defined parameters) that collectively cover the maximum number of nodes in all samples by solving the maximum conserved subnetwork cover problem (MCSC) via ILP. In both modes, cd-CAP runs in 2 steps. The first step computes a set of all "candidate" subnetworks (each with a distinct alteration pattern) with ≤*k* nodes, and which are shared by ≥*t* samples. However, the 2 modes differ in the second step: the first returns a single largest subnetwork, and the second returns *l* subnetworks collectively covering the maximum number of nodes from the set of candidate subnetworks.

Additionally cd-CAP provides the user the ability to add or relax some constraints on the subnetworks it identifies. Specifically, the user can ask cd-CAP to (i) return "colorful" subnetworks (i.e., subnetworks of nodes with ≥2 distinct colors) or (ii) allow up to a δ fraction of nodes in the subnetwork to have no alteration (as a result, not colored) in some of the samples that share the subnetwork.

We have applied cd-CAP—with both single and multi-subnetwork mode, with the basic setting (which only requires that each node has the same alteration type across the samples), as well as each of the possible additional options above, i.e., (i), (ii)—to The Cancer Genome Atlas (TCGA) breast adenocarcinoma (BRCA), colorectal adenocarcinoma (COAD), and glioblastoma multiforme (GBM) datasets. On these datasets, which collectively include >1,000 tumor samples, cd-CAP identified several connected subnetworks of interest, each exhibiting a specific gene alteration pattern across a large subset of samples.

In particular, cd-CAP results with the basic setting demonstrated that many of the largest highly conserved subnetworks within a tumor type solely consist of genes that have been subject to copy number gain, typically located on the same chromosomal arm and thus likely a result of a single, large-scale amplification. One of these subnetworks that cd-CAP observed (in about one-third of the COAD samples [29]) includes 9 genes in chromosomal arm 20q, which corresponds to a known amplification recurrent in colorectal tumors. Another copy number gain subnetwork cd-CAP observed in breast cancer samples corresponds to a recurrent large-scale amplification in chromosome 1 [30]. It is interesting to note that cd-CAP was able to rediscover these events without specific training.

Several additional subnetworks identified by cd-CAP solely consist of genes that are aberrantly expressed. Further analysis with option (ii) in the multi-subnetwork mode of cd-CAP revealed subnetworks that capture signaling pathways and processes critical for oncogenesis in a large fraction of tumors. We have also observed that the subnetworks identified through all different options of cd-CAP are associated with patients’ survival outcome and can hence be clinically important.

To assess the statistical significance of subnetworks discovered by cd-CAP in the single-subnetwork mode, we introduce for the first time a model in which likely interdependent events, in particular amplification or deletion of all genes in a single chromosome arm, are considered as a single event. Conventional models of gene amplification either consider each gene amplification independently [31] (this is the model we implicitly assume in our combinatorial optimization formulations, giving a lower bound on the true *P*-value) or assume that each amplification can involve >1 gene (forming a subsequent sequence of genes) but with the added assumption that the original gene structure is not altered and the duplications occur in some orthogonal "dimension" [32–34]. Both models have their assumptions that do not hold in reality but are motivated by computational constraints: inferring the evolutionary history of a genome with arbitrary duplications (that convert one string to another, longer string, by copying arbitrary substrings to arbitrary destinations) is an NP-hard problem (and is difficult to solve even approximately) [35,36]. By considering all copy number gain or loss events in the same chromosomal arm as a single event, we are, for the first time, able to compute an estimate that provides an empirical upper bound to the statistical significance (*P*-value) of the subnetworks discovered. (Note that this is not a true upper bound because a duplication event may involve both arms of a chromosome, but that would be extremely rare.) Through this upper bound, together with the lower bound above, we can sandwich the true *P*-value and thus the significance of our discovery.

## Methods

The Combinatorial Optimization Formulation section below describes the combinatorial optimization formulations used by cd-CAP to solve the problem of detecting conserved alteration patterns and all its abovementioned variants. The Algorithmic Details section describes implementation details for the 2 main steps of cd-CAP’s solution. The Additional Constraints and Parameter Options subsection describes the implementation details for the 2 variants on the constraints imposed by cd-CAP.

### Combinatorial optimization formulation

Consider an undirected and node-colored graph *G* = (*V, E*), representing the human gene or protein interaction network, with *n* nodes where *v_j_* ∈ *V* represent genes and *e* = (*v_h_*, *v_j_*) ∈ *E* represent interactions among the genes/proteins. A given sample/patient *P_i_* (among *m* samples in a cohort) has a specific coloring of *G*, namely *G_i_* = (*V, E, C_i_*), where each node *v_i, j_* (corresponding to node *v_j_* ∈ *V*) is colored with ≥1 possible colors to form the set *C_i, j_* (i.e., *C_i_* maps *v_i, j_* to a possibly empty subset of colors *C_i, j_*). Each color represents a distinct type of alteration harbored by a gene/protein: specific alteration types that we consider are somatic mutation (single-nucleotide alteration or short indel), copy number gain, copy number loss, or significant alteration in expression (this set of alterations can be trivially expanded to include genic structural alteration—micro-inversion or duplication, gene fusion, alternative splicing, methylation alteration, non-coding sequence alteration) observed in a gene or its protein product. Note that *C_i, j_* = ∅ implies that none of the alteration types we consider are observed at *v_i, j_*. Also note that given a node *v_j_*, its occurrences *v_i, j_* and $v_{i^{\prime },j}$, in respective samples *P_i_* and $P_i^{\prime }$, have ≥1 matching color if $C_{i,j}\cap C_{i^{\prime },j}\ne \emptyset$.

The main goal of cd-CAP is to identify conserved patterns of (i.e., identically colored) connected subnetworks across a subset of colored (sample) networks *G_i_*. Consider a connected subnetwork *T* = (*V_T_*, *E_T_*) of the interaction network *G*, where each node *v_j_* ∈ *V_T_* is assigned a single color *c_j_*. Such a colored subnetwork is said to be shared by a collection of patient networks {*G_i_*$\colon$*i* ∈ *I*} if the color *c_j_* assigned to each vertex *v_j_* is in the color set *C_i, j_* of each *v_i, j_*(*i* ∈ *I*), i.e., $c_j\in \bigcap _{i \in I} C_{i,j}$ for each *v_j_* ∈ *V_T_*. Note that *v_i, j_* is said to be covered by a colored subnetwork if that colored subnetwork is shared by *G_i_* (Fig. 1). Intuitively, a colored subnetwork represents a conserved pattern or a network motif.

In the single-subnetwork mode, cd-CAP solves MCSI, a specific combinatorial problem to identify conserved patterns of subnetworks. MCSI seeks to find the largest connected colored subnetwork *S* of the interaction network *G*, that occurs in exactly *t* (a user-specified number) samples $\mathcal {P}$, such that each node in *S* has the same color (assigned to it in *S*) in each sample $P_i\in \mathcal {P}$. Note that this formulation is orthogonal to that used in Bomersbach et al. [23] and Hristov and Singh [24], where the goal is to maximize the number of samples that share a fixed-size subnetwork. Unlike these formulations, MCSI admits a generalization of the a priori algorithm, which we use to solve it efficiently. Note that our formulation considers distinct types of mutations (as colors) in the conserved alteration patterns, another key improvement to alternative formulations used in the literature [23,24].

In the multi-subnetwork mode, on the other hand, cd-CAP aims to simultaneously identify multiple conserved subnetworks that are altered in a large number of samples. In particular, it may aim to cover all nodes *v_i, j_*, in all *m* input sample networks *G_i_*, with the smallest number of subnetworks *T* = (*V_T_*, *E_T_*) shared by ≥1 sample network. We refer to this combinatorial optimization problem as the minimum subgraph cover problem for (node) colored interaction networks (MSC-NCI). As shown below, cd-CAP solves a slightly more constrained variant of this problem in the multi-subnetwork mode.

The MSC-NCI problem, as described above, is parameter-free. However, in a realistic multi-omics cancer dataset, the number of genes far exceeds the number of samples represented. Under such conditions, the solution to the MSC-NCI problem will primarily include subnetworks that are large connected components that are shared by only 1 sample network. To account for this situation, we introduce the following parameters/constraints akin to those for the MCSI formulation: (i) we require that the nodes in each subnetwork have their assigned color shared by ≥*t* samples (in the remainder of the discussion, *t* is referred to as the "depth" of a subnetwork); and (ii) we require that each subnetwork returned contain ≤*k* nodes. Note that this variant of the problem is infeasible for certain cohorts (consider a particular node that has a unique color for a particular sample; clearly requirement [i] cannot be satisfied if *t* > 1). Even if there is a feasible solution, the requirement that each subnetwork in $\mathcal {T}$ be of size ≤*k* makes the problem NP-hard (the reduction is from the problem of determining whether *G* can be exactly partitioned into connected subnetworks, each with *k* nodes [37]). As a result (iii) we introduce 1 additional parameter, *l*, the maximum number of subnetworks (each of size ≤*k*, and which are color-conserved in ≥*t* samples), with the objective of covering the maximum number of nodes across all samples. We refer to the problem of identifying ≤*l* subnetworks of size ≤*k*, whose colors are conserved across ≥*t* samples, so as to maximize the total number of nodes in all these samples covered by these subnetworks, as the MCSC problem. cd-CAP solves MCSC via ILP in its multi-subnetwork mode.

### Algorithmic details

In this section we describe the detailed algorithmic framework of cd-CAP, which consists of 2 steps for both its single- and multi-subnetwork modes. The key insight as the basis of our algorithm is that in all instances of interest, only a limited number of genes are colored in comparison with the total number of nodes *nm*. This enables us to apply an exhaustive search method that is designed for association rule mining [28] to build a list of all candidate subnetworks exactly and efficiently (e.g., in comparison with the ILP or heuristic solutions in Bomersbach et al. [23] and Hristov and Singh [24]). Note that our exhaustive search method is an extension of the a priori algorithm, with the difference that we require the candidate subnetworks to maintain connectivity as they grow. As a result, we first compute the candidate subnetworks (each with a distinct alteration pattern) with ≤*k* nodes, and which are shared by ≥*t* samples in both modes. In the next step, in the single-subnetwork mode, cd-CAP simply returns the largest subnetwork among the candidate subnetworks, while in the multi-subnetwork mode it solves the MCSC on the set of candidate subnetworks via the ILP formulation below.

#### First step of cd-CAP: Generating candidate subnetworks

We generate the complete list of candidate subnetworks with minimum depth *t* by the use of the "anti-monotone" property [38]: if any subnetwork *S* has depth <*t*, then the depth of all of its supergraphs *S*′⊃*S* must be <*t*. This makes it possible to grow the set $\mathcal {S}$ of valid subnetworks comprehensively but without repetition (described as "optimal order of enumeration" in Maxwell et al. [39]) through the following breadth-first network growth strategy.

1. For every colored node *v_i, j_* and each of its colors *c*_ℓ_, we create a candidate subnetwork of size 1 (i.e., with single node) containing the node with color *c*_ℓ_. All samples in which the node is colored *c*_ℓ_ trivially share this subnetwork.
2. We inductively consider all candidate subnetworks of size *s* with the goal of growing them to subnetworks of size *s* + 1 as follows. For a given subnetwork *T* of size *s*, consider each neighboring node *u*. For each possible color $c_\ell ^{\prime }$ of *u*, we create a new candidate subnetwork of size *s* + 1 by extending *T* with *u—*with color $c_\ell ^{\prime }$. We maintain this subnetwork for the next inductive step only if the number of samples sharing this new subnetwork is ≥*t*; otherwise, we discard it.

The procedure is repeated until none of the subnetworks of size *s* + 1 covers ≥*t* samples (typically in the single-subnetwork mode), or until *s* = *k* − 1 (typically in the multi-subnetwork mode). Once the procedure terminates, the single-subnetwork mode simply returns all subnetworks constructed in the final iteration (of size *s*). The multi-subnetwork mode requires additional processing as described below. Note, however, that during the extension of *T* above, if the new node *u* does not reduce the number of samples sharing it, *T* becomes redundant and is not considered in the ILP formulation in the multi-subnetwork mode.

#### Second step of cd-CAP: Solving MCSC for multi-subnetwork mode

Given the universe $\mathcal {U}=\lbrace v_{i,j} \, | \, C_{i,j} \ne \emptyset \, , i = 1, \cdots , m; j = 1, \cdots , n\rbrace$, containing all the colored nodes in all the sample networks, and the collection of all subnetworks $\mathcal {S} = \lbrace T_i \, | \, T_i \, \text{is shared by $\ge t$ samples and contains $\le k$ nodes} \rbrace$, our goal is to identify up to *l* subnetworks from the set $\mathcal {S}$ that collectively contain the maximum possible number of elements of the universe $\mathcal {U}$.

After the list of all candidate subnetworks $\mathcal {S}$ is constructed (as described in the previous subsection), we represent the MCSC problem with the integer linear program below and solve it using IBM ILOG CPLEX or Gurobi. A binary variable *C*[*i, j*] corresponds to whether colored node *v_i, j_* was covered by ≥1 chosen subnetwork, and binary variable *X*[*i*] corresponds to whether colored candidate subnetwork *T_i_* was one of the chosen. Similarly $\mathcal {S}_{i,j}$ represents the set of all subnetworks of $\mathcal {S}$ that contain node *v_i, j_* properly colored in them.

**Table:** 

Maximize	$\displaystyle \sum _{v_{i,j} \in \mathcal {U}} C[i,j]$
	
such that	$\displaystyle \sum _{T_p \in \mathcal {S}_{i,j}} X[p] \ge C[i,j] \quad (\forall v_{i,j} \in \mathcal {U})$
	
	$\displaystyle \sum _{T_i \in \mathcal {S}} X[i] \le l.$

### Additional constraints and parameter options

In addition to the exactly conserved colored subnetworks obtained through the general MCSI or MCSC formulation as described above, cd-CAP offers the user the ability to add or relax constraints through new parameters, in both single- and multi-subnetwork mode.

- *Colorful conserved subnetworks*. In some of the datasets that we analyzed, certain variant types (i.e., colors) were dominant in the input to the extent that all subnetworks identified by our method had all nodes colored identically. By insisting that the identified subnetworks be ”colorful,” it is possible to, e.g., capture conserved genomic alterations and their impact on their interaction partners (form of expression alterations). For this purpose we introduce the notion of a colorful subnetwork, *T*, as a subnetwork that has ≥2 distinct colors represented in the coloring of its nodes, i.e., $c_\ell , c_h \in \bigcup _{v_j \in T} c_j \quad (c_\ell \ne c_h)$. To identify colorful subnetworks instead of arbitrary subnetworks, we update the first step of cd-CAP so that it specifically keeps track of colorful subnetworks (rather than all subnetworks) in each iteration; this is because any colorful network must contain a connected colorful subnetwork.
- *Subnetworks conserved within error rate δ*. To reduce the sensitivity of cd-CAP to noise (that emerges during the assignment of variant types to genes—due to limited precision of sequence or statistical analysis methods) in the input data, we provide the user the option to allow *errors* in identifying conserved subnetworks. For that, cd-CAP provides the user the option to specify an error rate δ that represents the fraction of nodes in a subnetwork *T* that can have no assigned color in any sample that shares *T*. We implemented this by updating the first step of cd-CAP so that it expands the set of samples that share each candidate subnetwork *T* to every other sample where *T* occurs with ≤δ|*T*| color omissions.

### Assessing the statistical and biological significance of the networks identified by cd-CAP

#### *Statistical significance of subnetworks identified by cd*-CAP

It is possible to assess the statistical significance of the subnetworks identified by cd-CAP by applying the conventional permutation test [13,23,27] on the color assignments of nodes, under the assumption that each gene is altered independently: let *C_i, j_* represent the set of colors assigned to a node *v_i, j_* and let $\mathcal {C}_i=\lbrace (v_{i, j}, C_{i,j})\rbrace$ represent the entire set of color assignments to nodes *v_i, j_* in network *G_i_*. We can obtain a random permutation of the color assignment $C_i^{\prime }$ by independently shuffling each color *c* ∈ ∪_*j*_*C_i, j_* across the nodes of *G_i_*, which results in an assignment of a new color set $C_{i,j}^{\prime }$ to each node *v_i, j_*, under the constraint that the total number of nodes with each color *c* is preserved. For a subnetwork *T* = (*V_T_*, *E_T_*) of size *k* covering *t* samples returned by cd-CAP in the single-subnetwork mode, we can carry out a permutation test as follows. First we generate a permuted color assignment (as described above) for each sample. Then we run cd-CAP in the single-subnetwork mode (possibly with the option [i] or [ii] as described in the previous section) and identify the largest subnetwork that covers ≥*t* samples. We repeat this sufficiently many (by default 1,000) times to compute *P*_1, *T*_, the number of times we end up with a subnetwork of size ≥*k* in *≥t* samples, normalized by the number of attempts. We can use *P*_1, *T*_ as an empirical *P*-value for subnetwork *T* of size *k*.

*P*_1, *T*_ forms an empirical lower bound for the *P*-value of *T* rather than an accurate estimate because it ignores the interdependencies among gene alteration events (i.e., node colors). In particular, whole-chromosome or chromosome arm–level copy number amplifications/deletions are commonly observed in cancer; such events must be reflected in the permutation test we use. To address this issue, we apply the following procedure to compute *P*_2, *T*_ as an empirical upper bound for the *P*-value of *T*, under the assumption that copy number alterations take place in whole chromosome arms. For a given color *E*, corresponding to either copy number gain or loss events, let *N_i, E_* denote the number of nodes with color *E* in *G_i_*. For each chromosomal arm $\mathcal {A}$, consider the set of nodes $V_{i,\mathcal {A}}$ that have been assigned ≥1 color in *G_i_*. Now we can reassign colors to vertices such that (i) colors *E* corresponding to copy number gain or loss are assigned to all genes in a chromosome arm simultaneously; specifically, the set of nodes $V_{i,\mathcal {A}}$ in a chromosome arm $\mathcal {A}$ are all assigned the same color *E* independently with probability *N_E_/*$\sum _{j} |C_{i,j}|$ (which guarantees that the expected number of nodes with color *E* in *G_i_* is preserved); (ii) the remaining colors (not related to copy number gain or loss) are assigned randomly to those nodes without a color assignment thus far (as described in the computation for *P*_1, *T*_). This process provides a new randomly permuted color assignment $C_i^{\prime \prime }$, which we use to obtain an empirical upper bound on the *P*-value of a subnetwork *T* discovered by cd-CAP. For that we perform this process simultaneously in all *G_i_* and check whether the largest subnetwork shared by ≥*t* samples exceeds the size of a subnetwork *T* (identified on the input dataset by cd-CAP). We repeat this process sufficiently many times and record the number of times the largest subnetwork obtained indeed exceeds the size of *T*; that value normalized by the number of times the process is executed is the value *P*_2, *T*_, the empirical upper bound on the *P*-value of *T*. The true *P*-value of *T* must be in the range [*P*_1, *T*_, *P*_2, *T*_] (provided that chromosome arms form the largest units of alteration).

#### Pathway enrichment analysis

We tested the set of genes in the subnetworks obtained by cd-CAP for enrichment against gene sets corresponding to pathways present in the Molecular Signature Database version 6.0 [40]. A hypergeometric test–based gene set enrichment analysis [40] was used for this purpose. A false discovery rate ≤0.01 was used as a threshold for identifying significantly enriched pathways.

#### Association between cd-CAP–identified subnetworks and patients’ survival outcome

To assess the association between each cd-CAP–identified subnetwork *T* with patients’ survival outcome, we used a risk score based on the (weighted) aggregate expression of all genes in the subnetwork *T*. The risk score (*S*) of a patient is defined as the sum of the normalized gene expression values in the subnetwork, each weighted by the estimated univariate Cox proportional-hazard regression coefficient [41], i.e., $S = \sum _{i}^{k} \beta _i x_{ij}$. Here *i* and *j* represent a gene and a patient, respectively; β_*i*_ is the coefficient of Cox regression for gene *i; x_ij_* is the normalized gene expression of gene *i* in patient *j*; and *k* is the number of genes in the subnetwork. The normalized gene expression values were fitted against overall survival time with living status as the censored event using univariate Cox proportional-hazards regression (exact method). On the basis of the risk score values, patients were stratified into 2 groups: low risk (patients with *S* < mean of *S*) and high risk (patients with *S* ≥ mean of *S*). Note that only those patients who are covered by the subnetwork are considered for the analysis above. In fact, with respect to survival outcomes, the set of patients covered by a subnetwork identified by cd-CAP would not necessarily differ from those who are not, because the latter set is likely to be highly heterogeneous with respect to cancer subtypes.

## Results

### Datasets and data processing

#### TCGA tumor variant data

We obtained somatic mutation, copy number aberration (CNA), and RNA sequencing–based gene expression data from 3 distinct cancer types: GBM [42], BRCA [43], and COAD [29] from TCGA datasets (detailed information can be found in Supplementary Section 1). In addition, we distinguish 4 commonly observed molecular subtypes (i.e., luminal A, luminal B, triple-negative/basal-like, and HER2-enriched) from the BRCA cohort. For each sample, we obtained the list of genes that harbor somatic mutations, CNAs, or are expression outliers as per below.

Somatic mutations. All non-silent variant calls that were identified by ≥1 tool among MUSE, MuTect2, SomaticSniper, and VarScan2 were considered.

CNAs. CNA segmented data from the National Cancer Institute Genomic Data Commons were further processed using Nexus Copy Number Discovery Edition version 9.0 (BioDiscovery, Inc., El Segundo, CA) to identify aberrant regions in the genome. We restricted our analysis to the most confident CNA calls, selecting only those genes with high copy gain or homozygous copy loss.

Expression outliers. We used HTSeq-FPKM-UQ normalized RNA sequencing expression data to which we applied the generalized extreme studentized deviate (GESD) test [44]. In particular, we used the GESD test to compare the transcriptome profile of each tumor sample (one at a time) with that from a number of available normal samples. For each gene, if the tumor sample was identified as the most extremely deviated sample (using critical value α = 0.1), the corresponding gene was marked as an expression outlier for that tumor sample. This procedure was repeated for every tumor sample. Finally, comparing the tumor expression profile of these outlier genes with the normal samples, their up- or downregulation expression patterns were determined.

#### Interaction networks

We used the following human protein interaction networks in the identification of the most significant subnetworks specific to the cancer types mentioned above: (i) STRING version 10 [45] protein interaction network, which contains high-confidence functional PPIs. Self-loops and interactions with missing HUGO Gene Nomenclature Committee symbols were discarded and interaction scores were normalized (divided by 1,000) to obtain a reliability score in the range [0, 1]. Only high-confidence interactions with a combined score of ≥0.9 were selected. (ii) STRING network with only experimentally verified edges. (iii) Human Protein Reference Database (HPRD) version 9 [46]. (iv) REACTOME version 2015 [47].

### Maximal colored subnetworks across cancer types

We used cd-CAP to solve the MCSI problem exactly on each of the protein interaction networks we considered on all cancer types, for every feasible value of network depth. As can be easily observed, the depth and the size of the identified subnetwork are inversely related. We say that a network depth value is feasible if (i) the depth is ≥10% of the cohort size, (ii) the maximum network size for that depth is ≥3, and (iii) the number of "candidate" subnetworks is ≤2 million per iteration when running cd-CAP for that depth.

**Figure 2:**
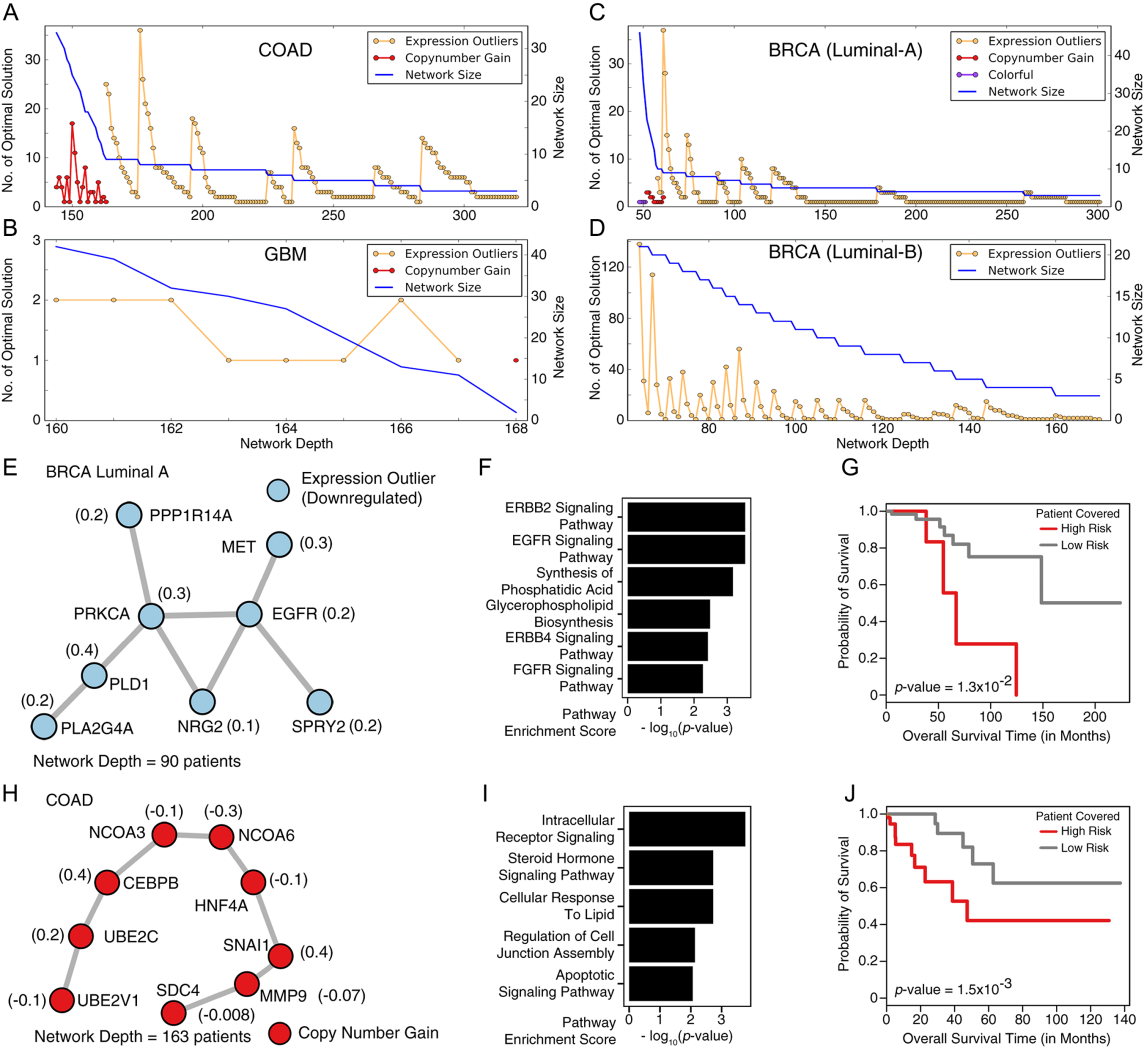
**Conserved colored subnetworks**. (A–D) Number of maximal solutions and the size of the conserved colored subnetwork obtained using the MCSI formulation, as a function of network depth *t*, in each of 4 cancer types analyzed, on STRING version 10 (with high-confidence edges) PPI network. The horizontal axis denotes the depth (number of patients) of the network. For the blue curve, the vertical axis denotes the maximum possible network size (in terms of the number of nodes) and thus it is strictly non-increasing by definition. For the curves with different colors, the vertical axis denotes the number of distinct networks with network size equal to that indicated by the blue curve. As can be seen, the red curves depict networks where all nodes have a copy number gain, the yellow curves depict networks where all nodes are expression outliers, and purple curves depict colorful networks (with ≥2 distinct colors). A total of 41 subnetworks across all cancer types (10 COAD, 4 GBM, 11 luminal A, and 16 luminal B) correspond to the ends of "valleys" in the color plots and were further analyzed. Two of the most interesting ones are provided here, both of which are uni-colored. The number in parentheses next to each node represents the univariate Cox proportional-hazards regression coefficient estimated for each gene, used as its weight in the risk score calculation to stratify patients into 2 distinct risk groups. (See Methods section for details). (E–G) One of the 11 maximal colored subnetworks identified in the BRCA luminal A dataset. It consists solely of downregulated expression outlier genes and has depth 90 (patients). (E) The colored subnetwork (with 8 nodes) topology. (F) Pathways dysregulated by alterations harbored by the genes in the subnetwork; these genes are involved in the epidermal growth factor receptor (EGFR), ERBB2, and fibroblast growth factor receptor (FGFR) signaling pathways. (G) Kaplan-Meier plot showing the significant association of the subnetwork, with patients’ clinical outcome. Patients "covered" by the subnetwork were stratified into 2 groups, high risk (8 patients) and low risk (82 patients), on the basis of their gene expression levels (see Methods for details). (H–J) One of the 10 maximal colored subnetworks identified in the COAD dataset; it consists solely of copy number amplified genes and has a depth of 163 (patients). Genes in this subnetwork belong to the same chromosomal locus 20q13. (H) The colored subnetwork (with 9 nodes) topology. (I) Pathways dysregulated by the alterations harbored by the genes in the subnetwork; these genes are involved in signal transduction and the apoptotic process. (J) Kaplan-Meier plot showing the significant association of the subnetwork with patients’ clinical outcomes (73 high risk vs 83 low risk patients).

The number of maximal solutions of cd-CAP as a function of feasible network depth for each cancer type (COAD, GBM, BRCA luminal A, and BRCA luminal B) is shown in Fig. 2A–D on STRING version 10 PPI network with high-confidence edges (see Supplementary Figs 2–5 for the results on alternative PPI networks). In general, for a fixed network size, the number of distinct networks of that size decreases as the network depth increases. One can observe that the ends of "valleys" in the colored plots in Fig. 2A–D correspond to the largest depth that can be obtained for a given subnetwork size.

In the remainder of the article we focus only on the single colored subnetwork of each given size that has the maximum possible depth (corresponding to the ends of the "valleys" in the plots). (If, for a given subnetwork size and the corresponding maximal depth, cd-CAP returns >1 subnetwork, they are ignored.)

Many of the subnetworks that we focused on, especially those with large depth, only consisted of expression outlier genes (typically all upregulated or all downregulated) (Fig. 2A–D), across all 4 cancer types. In the luminal A dataset, for example, cd-CAP identified a subnetwork of 8 downregulated genes with a network depth of 90 (Fig. 2E), consisting of genes *EGFR, PRKCA, SPRY2*, and *NRG2*, known to be involved in the EGFR/ERBB2/ERBB4 signaling pathways (Fig. 2F). *EGFR* is an important driver gene involved in the progression of breast tumors to advanced forms [48], and its altered expression is observed in a number of breast cancer cases [30]. The subnetwork also included *MET*, another well-known oncogene [49], and is enriched for members of the Ras signaling pathway, which is also known for its role in oncogenesis and mediating cancer phenotypes such as overproliferation [50].

cd-CAP additionally identified some (uni-colored) copy number gain networks, typically with lower depth: a prominent example is in the COAD dataset with depth 163 (out of 463 patients in the cohort). This network forms the core of larger (maximal) subnetworks that cd-CAP identifies for lower depth values; it corresponds to a copy number gain of the chromosomal arm 20q—a well-known CNA pattern highly specific to COAD tumors [29]. Another subnetwork that cd-CAP identified in 15% of the 422 BRCA luminal A samples corresponds to a copy number gain on chromosome 1 that is again a known aberration associated with breast cancer [30].

Note that cd-CAP also identified several multi-colored subnetworks. The benefits of cd-CAP’s ability to identify multi-colored subnetworks are demonstrated in Supplementary Fig. 1, which summarizes the results of a comparison between cd-CAP and a limited version of cd-CAP that does not differentiate mutation types. The figure shows that, especially in COAD and GBM, the survival outcomes of samples that include the cd-CAP–identified subnetworks differ significantly from those sameples that do not include such subnetworks. In the BRCA dataset, because all subnetworks of interest involve differentially expressed genes, the difference between survival outcomes is insignificant.

A complete list of subnetworks of focus (from STRING version 10 with high-confidence edges), across all cancer datasets, is provided in Supplementary Table 2. For each of these subnetworks, and for each patient covered by a particular subnetwork, we calculated a risk score defined as a linear combination of the normalized gene expression values of the genes in the subnetwork weighted by their estimated univariate Cox proportional-hazards regression coefficients (see Methods section for details). On the basis of the risk score values, the patients covered by the subnetwork were stratified into 2 risk groups (high and low risk).

The expression outlier subnetwork that we mentioned above for the luminal A dataset was the most significant among all subnetworks identified in this dataset (Fig. 2G). The patients in the high-risk group have poor overall survival outcome, suggesting the clinical importance of the subnetwork identified by cd-CAP.

Another copy number gain subnetwork shared among 163 patients in the COAD dataset (Fig. 2H) was composed of genes from chromosome locus 20q13, likely indicating a single chromosomal amplification event. Intriguingly, these genes form a linear structure on the protein interaction network. Among them is a group of functionally related genes consisting of transcription factors and their regulators (genes *CEBPB, NCOA3, NCOA6, UBE2C, UBE2V1*), which are known to be involved in the intracellular receptor signaling pathway (Fig. 2I). *CEBPB* and *UBE2C* are also involved in the regulation of the cell cycle [51]. At the other end of the linear subnetwork, there are *MMP9* and *SDC4*, established mediators of cancer invasion and apoptosis [52,53]. We also confirmed that these genes are highly predictive of patients’ survival outcome (Fig. 2J). All these results seem to support the finding that cd-CAP–identified subnetworks are functionally important with potential clinical relevance.

### Maximal colorful subnetworks across cancer types

**Figure 3:**
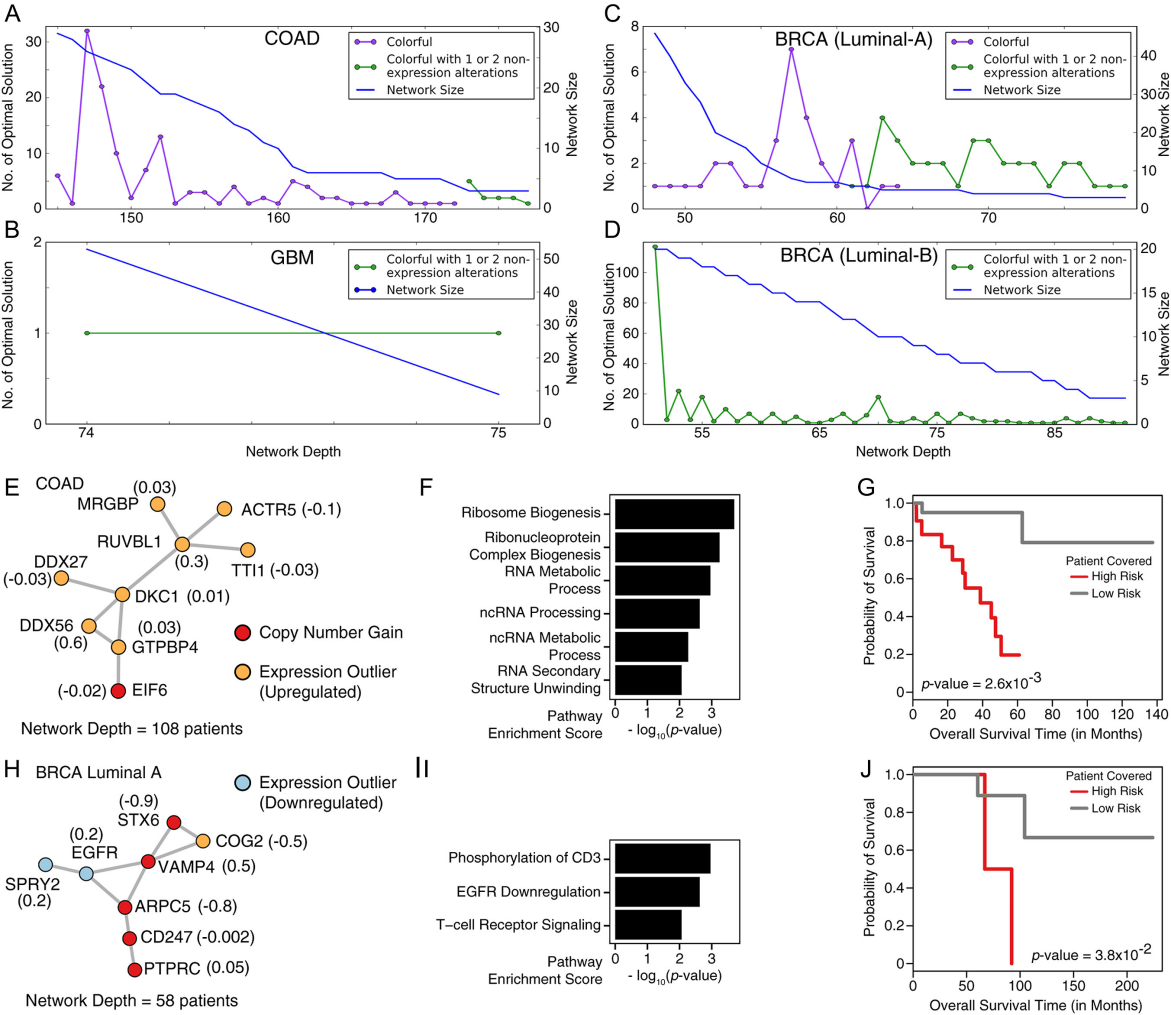
Colorful maximal subnetworks. (A–D) Number of maximal solutions and the size of the conserved colorful subnetwork obtained using the MCSI formulation, as a function of network depth *t*, in each of the cancer types analyzed on the STRING version 10 (high-confidence edges) PPI network. The horizontal axis denotes the depth (number of patients) of the network. For the blue curves, the vertical axis denotes the maximum possible network size (in terms of the number of nodes) and thus it is strictly non-increasing by definition. For the curves with different colors, the vertical axis denotes the number of distinct networks with network size equal to that indicated by the blue curve. As can be seen, the purple curves depict colorful subnetworks and the green curves depict networks that include 1–2 nodes that are not expression outliers. A similar analysis was performed on the STRING version 10 (experimentally validated edges), REACTOME, and HPRD PPI networks. A total of 104 colorful subnetworks corresponding to the ends of "valleys" of the plots were identified across the 4 cancer types in all the above PPI networks. Two of the most interesting ones are provided here. The number in parentheses next to each node represents the univariate Cox proportional-hazards regression coefficient estimated for that gene, used as its weight in the risk score calculation to stratify the patients into 2 distinct risk groups (see Methods section for details). (E–G) One of the maximal colorful subnetworks identified in the COAD dataset, consisting of ≤2 non-expression outlier (for this case copy number gain) genes, with depth 108 (patients). (E) The colored subnetwork (with 9 nodes) topology, obtained from STRING version 10 (with experimentally validated edges) PPI network. (F) Pathways dysregulated by alterations harbored by the genes in the subnetwork; these genes are involved in ribosome biogenesis and RNA processing. (G) Kaplan-Meier plot showing the significant association of the subnetwork with patients’ clinical outcomes (59 high-risk vs 47 low-risk patients). (H–J) One of the maximal colorful subnetworks identified in the luminal A dataset with no color restrictions, with depth of 58 (patients). (H) The colored subnetwork (with 8 nodes) topology, obtained in the REACTOME PPI network. (I) Pathways dysregulated by the alterations harbored by the genes in the subnetwork. (J) Kaplan-Meier plot showing the significant association of the subnetwork with patients’ clinical outcomes (30 high-risk vs 30 low-risk patients).

We used cd-CAP to solve the maximum conserved colorful subnetwork identification problem in each of the 4 protein interaction networks and each cancer type that we considered (see section Additional Constraints and Parameter Options for details). Again, cd-CAP was run with every feasible value (as defined above) of network depth. The number of maximal solutions of cd-CAP as a function of network depth for each cancer type (COAD, GBM, BRCA luminal A, and BRCA luminal B) is shown in Fig. 3 A–D on the STRING version 10 PPI network with high-confidence edges (see Supplementary Figs 2–4 for the results on alternative PPI networks). Note that we pay special attention to subnetworks with ≥1 sequence-altered gene (i.e., a gene that is somatically mutated or copy number altered) because the sequence alteration(s) may explain expression level changes in the remaining genes of the subnetwork (Fig. 3E provides such an example).

One such COAD subnetwork is composed of several overexpressed genes and 1 copy number gain gene, covering 108 patients (Fig. 3E). This subnetwork is mainly enriched for genes involved in ribosome biogenesis (Fig. 3F). Cancer has long been known to place an increased demand on ribosome biogenesis [54], and increased ribosome generation has been reported to contribute to cancer development [55]. The biological relevance of this subnetwork is also supported by survival analysis, which shows a strong differentiation between the high-risk and low-risk groups; see Fig. 3G.

Another subnetwork that we observed in 58 BRCA luminal A samples consists of 4 copy number gain genes, an overexpressed gene, and 2 underexpressed genes, including *EGFR* (Fig. 3H). All copy number gain genes and the overexpressed gene are located in chromosome 1q, commonly reported in breast cancer [30]. The subnetwork involves an interesting combination of the downregulation of the cancer gene *EGFR* and the amplification of a group of genes involved in T-cell receptor signaling (*PTPRC, CD247*, and *ARPC5*; see Fig. 3I). Thus, we may surmise that the covered population of patients potentially have a relatively low cancer proliferation index with higher anti-tumor immune response, which can be highly relevant indicators with respect to clinical outcome. Indeed, this subnetwork is significantly associated with patients’ survival (Fig. 3J).

### Multiple-subnetwork analysis across cancer types

We next sought to detect up to 5 subnetworks per cancer type that collectively cover the maximum possible number of colored nodes by solving the MCSC problem on the STRING version 10.5 network (with experimentally validated edges). The subnetwork extension error rate was set to 20%, and we restricted the search space to subnetworks that do not consist only of expression outlier nodes, in order to obtain what we believe to be more biologically interesting results. The network depth *t* was chosen for each dataset in a way that made it possible to construct all candidate subnetworks of maximum possible size while keeping the total number of candidate subnetworks <2 × 10^6^, making the problem solvable in a reasonable amount of time. We set *t* to 69 (15% of the patients), 62 (10% of the patients), and 110 (10% of the patients) respectively, for the COAD, GBM, and BRCA datasets. Supplementary Table 1 shows the size, per sample depth, and the coloring of the nodes in the resulting subnetworks.

We note that the subnetworks identified in the GBM dataset had the lowest depth (10–15% of the samples). The COAD and BRCA datasets, on the other hand, have much larger depth (respectively, 30–48% and 15–32% of the samples). Smaller subnetworks of the GBM dataset solely consist of copy number gain genes on chromosome 7q, a known amplification in GBM [56]. The 2 large subnetworks each contain a single gene with copy number gain (*SEC61G* and *EGFR*, respectively) accompanied by several of overexpressed genes. The BRCA dataset exhibits a similar pattern: each of the 4 large subnetworks contain a single copy number gain gene from chromosome 8q (*NSMCE2* in 1 and *MYC* in the remaining 3 subnetworks). Subnetworks detected in the COAD dataset were much more colorful and recurrently conserved in a larger fraction of samples than those in the other datasets. All genes with copy number gain are located in chromosome 20q.

**Figure 4:**
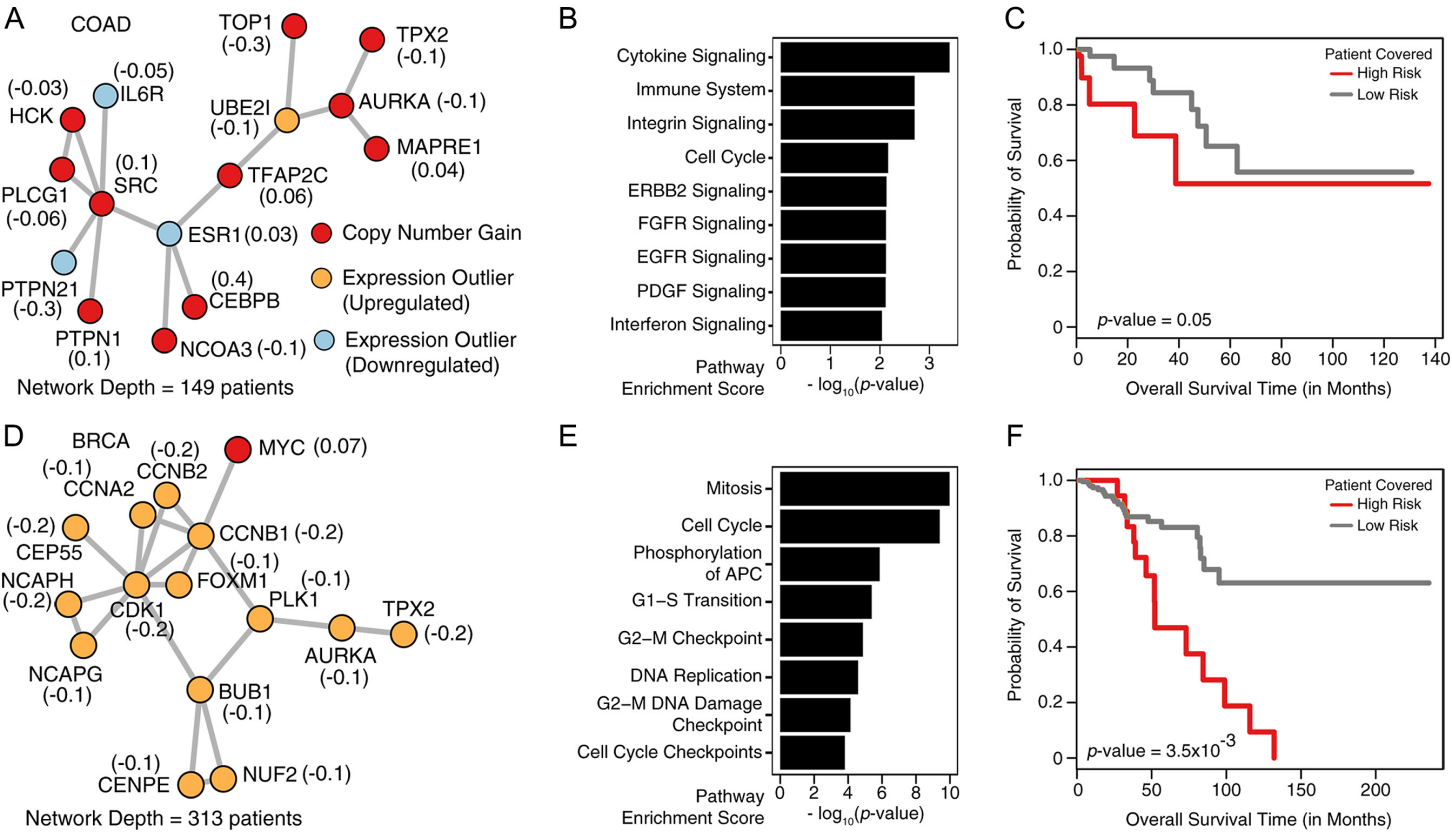
Multiple Subnetwork Analysis. Two of the largest subnetworks identified across the COAD, GBM, and BRCA datasets (5 networks were identified per cancer type) through the MCSC formulation of cd-CAP on the STRING version 10.5 (with experimentally validated edges) PPI network. The number in parentheses next to each node represents the univariate Cox proportional-hazards regression coefficient estimated for that gene, used as its weight in the risk score calculation to stratify the patients into 2 distinct risk groups (see Methods section for details). (A–C) The largest of the 5 COAD subnetworks with a network depth of 149 (patients). (A) The subnetwork topology (with 15 nodes). (B) Pathways dysregulated by alterations harbored by the genes in the subnetwork. (C) Kaplan-Meier plot showing the significant association of the subnetwork with patients’ clinical outcomes (69 high-risk vs 78 low-risk patients). (D-F) The largest of the 5 BRCA subnetworks with a network depth of 313 (patients). (D) The subnetwork topology (with 15 nodes). (E) Pathways dysregulated by the alterations harbored by the genes in the subnetwork. (F) Kaplan-Meier plot showing the significant association of the subnetwork with patients’ clinical outcomes (33 high-risk vs 278 low-risk patients).

We identified a subnetwork with 15 nodes (11 genes with copy number gain, 1 overexpressed, and 3 underexpressed genes) in 149 COAD patients (Fig. 4A). All 11 copy number gain genes belong to chromosome 20q. *IL6R, PLCG1, PTPN1*, and *HCK* are involved in cytokine/interferon signaling to activate immune cells to counter proliferating tumor cells [57] (Fig. 4B). *UBE2I, AURKA*, and *MAPRE1* are involved in cell cycle processes. This subnetwork was found to be associated with patients’ survival outcome (Fig. 4C).

We identified another subnetwork with 15 nodes (14 overexpressed and 1 copy number gain genes) in 313 breast cancer patients (Fig. 4D). Genes in this subnetwork are involved in cell cycle processes (Fig. 4 E). In particular the cell cycle checkpoint processes were dysregulated, which is known to drive tumor initiation processes [58]. The subnetwork was found to be associated with patients’ survival outcomes (Fig. 4 F), suggesting its potential clinical relevance.

### Empirical *P*-value estimates confirm the significance of cd-CAP–identified networks

To evaluate the significance of cd-CAP’s findings, we performed the permutation test described earlier 1,000 times on each cancer type for each possible setting of subnetwork constraints. Supplementary Tables 2–3 and Figure 6 demonstrate the distribution of the empirical *P*-value upper bound estimates with the STRING 10 (high-confidence edges) PPI network, while the lower bound results look similar to what is presented in the figure and thus are omitted. In the permutation tests all cd-CAP–identified subnetworks (without additional constraints) of size 2–5 were composed solely of expression-altered genes; in contrast, there are several larger copy number variation–rich subnetworks observed in the TCGA COAD dataset and others, further confirming the significance of our findings. Colorful subnetworks presented in Fig. 3 are even less likely to occur at random (we therefore omit empirical *P*-value estimates for the networks in Fig. 3).

## Discussion

In this article we introduce a novel combinatorial framework and an associated tool named cd-CAP that can identify (≥1) subnetworks of an interaction network where genes exhibit conserved alteration patterns across many tumor samples. Compared with the state-of-the-art methods (e.g., [22,24]), cd-CAP differentiates alteration types associated with each gene (rather than relying on binary information of a gene being altered or not) and simultaneously detects multiple alteration type conserved subnetworks.

cd-CAP provides the user with 2 major options. (i) In single-subnetwork mode, it computes the largest colored subnetwork that appears in ≥*t* samples. This option exhibits significant speed advantage over available ILP-based approaches; its a priori–based algorithmic formulation allows flexible integration of special constraints (on maximal subnetworks)—not only simplifying complicated ILP constraints but also further reducing the number of candidate subnetworks in iteration steps (a good example for this is the "colorful conserved subnetworks" as introduced in section Additional Constraints and Parameter Options). However, the identified subnetworks are required to be *conserved*; i.e., each node only admits 1 alteration type among the samples sharing it (although we have relaxed constraints that allow each sample to have a few nodes without any alterations, i.e., colors). In the future, we may be able to extend the definition of a network to include nodes with color mismatches (e.g., according to the definition in [21] or [22]) with a modification to cd-CAP’s candidate subnetwork generation algorithm. (ii) In multi-subnetwork mode, it solves the MCSC problem to cover the maximum number of nodes in all samples with ≤*l* colored subnetworks (*l* is user defined) via ILP. In the future we aim to refine the MCSC formulation with a reduced number of parameters and hope to develop exact or approximate solutions.

Subnetworks identified by cd-CAP in COAD, GBM, and BRCA datasets from TCGA are typically enriched with genes harboring gene expression alterations or copy number gain. Notably, we observed that genes in subnetworks with copy number amplification are universally located in the same chromosomal locus. Many of these genes have known interactions and are functionally similar, demonstrating the ability of cd-CAP to capture functionally active subnetworks, conserved across a large number of tumor samples. These subnetworks seem to overlap with pathways critical for oncogenesis. In the datasets analyzed, we observed cell cycle, apoptosis, RNA processing, and immune system processes that are known to be dysregulated in a large fraction of tumors. cd-CAP also captured subnetworks relevant to the EGFR/ERBB2 signaling pathways, which have distinct expression patterns in specific subtypes of breast cancer [30,59]. Survival analysis of cd-CAP–identified subnetworks also highlighted their potential for clinical relevance. In the future, it may be possible to use tissue-specific interaction data (such as [60] or [61]) to capture subnetworks with gene interactions that are more relevant to a specific cancer and tissue type.

## Availability of supporting data and materials

Supporting data and an archival copy of the code are available via the GigaScience database, GigaDB [62].

## Availability of source code and requirements

- Project name: cd-CAP
- Project home page: https://github.com/ehodzic/cd-CAP
- Operating system(s): Platform independent
- Programming language: C++
- Other requirements: make (version 3.81 or higher), g++ (GCC version 4.1.2 or higher), and IBM ILOG CPLEX Optimization Studio
- License: MIT License
- SciCrunch RRID: SCR_016843

## Additional files

Supplementary file is cd_CAP_sup.pdf.

## Abbreviations

BRCA: breast adenocarcinoma; cd-CAP: combinatorial detection of conserved alteration patterns; COAD: colorectal adenocarcinoma; CNA: copy number aberrations; GBM: glioblastoma multiforme; GESD: generalized extreme studentized deviate; HPRD: Human Protein Reference Database; ILP: integer linear programming; MCSI: maximum conserved subnetwork identification problem; MSC-NCI: minimum subgraph cover problem for (node) colored interaction networks; MCSC: maximum conserved subnetwork coverage problem; PCAWG: Pan Cancer Analysis of Whole Genomes; PPI: protein-protein interaction; TCGA: The Cancer Genome Atlas.

## Competing interests

The authors declare that they have no competing interests.

## Funding

This project was funded by the following: S.C.S. was supported in part by National Science Foundation grant CCF-1619081, National Institutes of Health grant GM108348, and the Indiana University Grand Challenges Program, Precision Health Initiative. R.S. is supported by Mitacs Accelerate Awards. E.H. is supported by NSERC-CREATE Computational Methods for the Analysis of the Diversity and Dynamics of Genomes (MADD-Gen) program.

## Authors' contributions

S.C.S. conceived and directed the project. E.H., R.S., K.Z., and S.C.S. developed the algorithm. E.H. and K.Z. developed the cd-CAP software. E.H., R.S., K.Z., and K.C. performed the data analysis. R.S, K.C., and C.C.C. helped in biological interpretation of the results. All authors contributed to the preparation and revision of the manuscript.

## Supplementary Material

giga-d-18-00320_original_submission.pdf

giga-d-18-00320_revision_1.pdf

giga-d-18-00320_revision_2.pdf

giga-d-18-00320_revision_3.pdf

giga-d-18-00320_revision_4.pdf

response_to_reviewer_comments_original_submission.pdf

response_to_reviewer_comments_revision_1.pdf

response_to_reviewer_comments_revision_2.pdf

response_to_reviewer_comments_revision_3.pdf

reviewer_1_report_original_submission -- Aaron McKenna 8/27/2018 Reviewed

reviewer_1_report_revision_1 -- Aaron McKenna 12/15/2018 Reviewed

reviewer_2_report_original_submission -- Evan Paull 10/2/2018 Reviewed

Supplemental Files
