# Supplementary material for: Combinatorial Detection of Conserved Alteration Patterns for Identifying Cancer Subnetworks
Source: Gigascience. 2019 Apr 11;8(4):giz024. doi: 10.1093/gigascience/giz024 (PMC6458499; doi:10.1093/gigascience/giz024)
Supplement: giga-d-18-00320_original_submission.pdf [file giz024_giga-d-18-00320_original_submission.pdf]

GigaScience

Combinatorial Detection of Conserved Alteration Patterns for Identifying Cancer Subnetworks

--Manuscript Draft--

**Manuscript Number:**	GIGA-D-18-00320
**Full Title:**	Combinatorial Detection of Conserved Alteration Patterns for Identifying Cancer Subnetworks
**Article Type:**	Research
**Funding Information:**	Indiana University Bloomington (Precision Health Initiative) — Dr. Cenk Sahinalp
**Abstract:**	Motivation: Advances in large scale tumor sequencing have lead to an understanding that there are combinations of genomic and transcriptomic alterations specific to tumor types, shared across many patients. Unfortunately, computational identification of functionally meaningful shared alteration patterns, impacting gene/protein interaction subnetworks has proven to be challenging.<br><br>Results: We introduce a novel combinatorial method, cd-CAP, for simultaneous detection of connected subnetworks of an interaction network where genes exhibit conserved alteration patterns across tumor samples. Our method differentiates distinct alteration types associated with each gene (rather than relying on binary information of a gene being altered or not), and simultaneously detects multiple alteration profile conserved subnetworks. In a number of TCGA data sets, cd-CAP identified large biologically significant subnetworks with conserved alteration patterns, shared across many tumor samples.<br><br>Availability: https://github.com/ehodzic/cd-CAP
**Corresponding Author:**	Kaiyuan Zhu<br>Indiana University Bloomington<br>Bloomington, IN UNITED STATES
**Corresponding Author Secondary Information:**	
**Corresponding Author's Institution:**	Indiana University Bloomington
**Corresponding Author's Secondary Institution:**	
**First Author:**	Ermin Hodzic
**First Author Secondary Information:**	
**Order of Authors:**	Ermin Hodzic
	Raunak Shrestha
	Kaiyuan Zhu
	Kuoyuan Cheng
	Colin Collins
	Cenk Sahinalp
**Order of Authors Secondary Information:**	
**Additional Information:**	
**Question**	**Response**
Are you submitting this manuscript to a special series or article collection?	No
**Experimental design and statistics**	Yes

Powered by Editorial Manager® and ProduXion Manager® from Aries Systems Corporation

Full details of the experimental design and statistical methods used should be given in the Methods section, as detailed in our Minimum Standards Reporting Checklist. Information essential to interpreting the data presented should be made available in the figure legends.<br><br>Have you included all the information requested in your manuscript?	
**Resources**<br><br>A description of all resources used, including antibodies, cell lines, animals and software tools, with enough information to allow them to be uniquely identified, should be included in the Methods section. Authors are strongly encouraged to cite Research Resource Identifiers (RRIDs) for antibodies, model organisms and tools, where possible.<br><br>Have you included the information requested as detailed in our Minimum Standards Reporting Checklist?	Yes
**Availability of data and materials**<br><br>All datasets and code on which the conclusions of the paper rely must be either included in your submission or deposited in publicly available repositories (where available and ethically appropriate), referencing such data using a unique identifier in the references and in the "Availability of Data and Materials" section of your manuscript.<br><br>Have you have met the above requirement as detailed in our Minimum Standards Reporting Checklist?	Yes

Manuscript Click here to download Manuscript cd_CAP_main.pdf

Combinatorial Detection of Conserved Alteration Patterns for Identifying Cancer Subnetworks

Ermin Hodzic*,1, 2, Raunak Shrestha*,1, 3, Kaiyuan Zhu*,4, Kuoyuan Cheng[5], Colin C. Collins[1], and S. Cenk Sahinalp†,4

[1]Laboratory for Advanced Genome Analysis, Vancouver Prostate Centre, Vancouver, BC, Canada,
[2]School of Computing Science, Simon Fraser University, Burnaby, BC, Canada,
[3]Bioinformatics Training Program, University of British Columbia, Vancouver, BC, Canada,
[4]Department of Computer Science, Indiana University, Bloomington, IN, USA,
[5]Center for Bioinformatics and Computational Biology, University of Maryland, College Park, MD, USA.

Abstract

Motivation: Advances in large scale tumor sequencing have lead to an understanding that there are combinations of genomic and transcriptomic alterations specific to tumor types, shared across many patients. Unfortunately, computational identification of functionally meaningful shared alteration patterns, impacting gene/protein interaction subnetworks has proven to be challenging.

Results: We introduce a novel combinatorial method, cd-CAP, for simultaneous detection of connected subnetworks of an interaction network where genes exhibit conserved alteration patterns across tumor samples. Our method differentiates distinct alteration types associated with each gene (rather than relying on binary information of a gene being altered or not), and simultaneously detects multiple alteration profile conserved subnetworks. In a number of TCGA data sets, cd-CAP identified large biologically significant subnetworks with conserved alteration patterns, shared across many tumor samples.

Availability: `https://github.com/ehodzic/cd-CAP`

1 Introduction

Recent large scale tumor sequencing projects such as PCAWG (Pan Cancer Analysis of Whole Genomes) have revealed multitude of somatic genomic, transcriptomic, proteomic and epigenomic alterations across cancer types. However, a tumor is likely driven by selected few alterations that provide evolutionary advantage to the tumor, hence called "driver" alterations [1]. Distinguishing driver alterations from functionally inconsequential random "passenger" alterations is critical for therapeutic development and cancer treatment.

It is well evident that, except for few cases, cancers are often driven by multiple driver genes [2, 3]. Whereas emergence of alterations is likely a consequence of endogenous or exogenous mutagen

*Joint first authors - listed alphabetically
†Corresponding author: cenksahi@indiana.edu

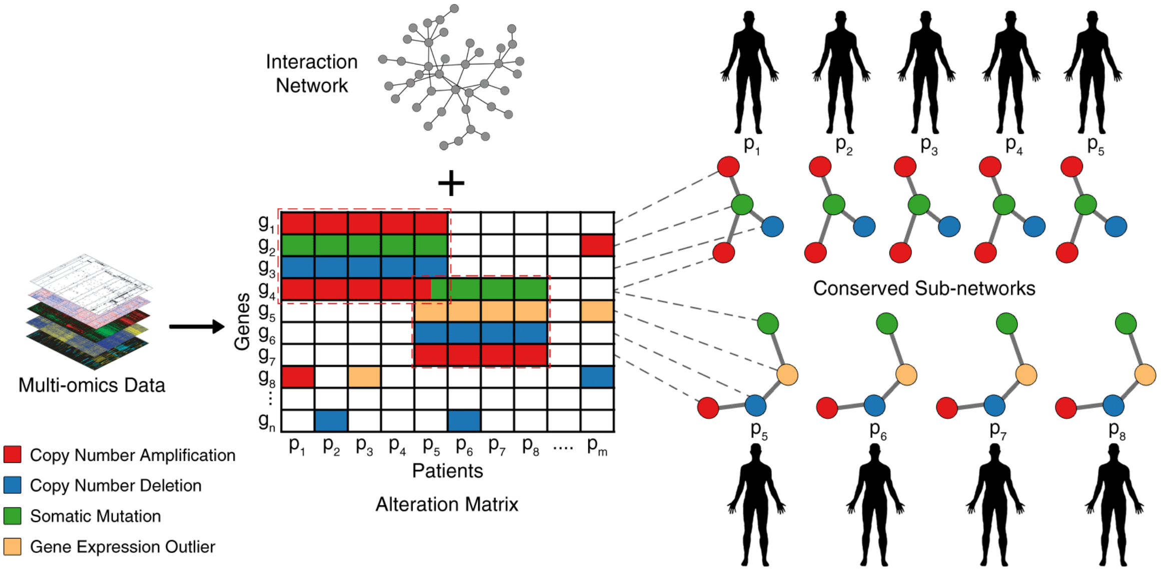

Figure 1: Schematic Overview of our framework. Multi-omics alteration profiles of a cohort of tumor samples are identified using appropriate bioinformatics tools. The alteration information is combined with gene-level information in the form of a sample-gene alteration matrix. Each alteration type is assigned a distinct color. Using a (signaling) interaction network, cd-CAP identifies subnetworks with conserved alteration patterns.

exposures [4], their evolutionary selection depends on the functional role of the affected genes [1] and synergistic combinations of different alterations. For example, *TMPRSS2-ERG* gene fusion is considered as an early driver event in almost half of prostate cancer cases, and it often co-exists with copy-number deletions of *PTEN* as well as *NKX3-1* to drive cancer progression [5, 6, 7]. Recently, concomitant deletion of four cancer genes - *BAP1*, *SETD2*, *PBRM1*, and *SMARCC1* in chromosome locus 3p21 has been identified as a driver event in a fraction of clear cell renal cell carcinoma (ccRCC) [8], uveal melanoma [9], and mesotheliomas [10]. These genes are involved in chromatin remodeling process, and their loss further impairs DNA damage repair pathway in the aberrant tumors [9].

Co-occurring alterations might be evolutionary selected because alteration in one gene might enhance the deleterious effect of the other [11]. Such co-selected genes are often a part of a functionally interacting driver subnetwork (i.e. module or pathway) that are observed together in the same tumor, and define its phenotype. In fact, as demonstrated by the Pan Cancer Analysis of Whole Genomes (PCAWG) Project [12] and other large scale sequencing efforts, co-occurring genomic and transcriptomic alterations in specific tumor types are commonly shared across a large fraction of patients. Thus efficient computational methods that can identify large subsets of functionally interacting (genomic or transcriptomic) alterations, highly conserved across specific tumor types, are in high demand.

Recently, a number of computational methods have been developed to identify recurrent genomic (as well as transcriptomic) alteration patters across tumor samples. Some of these methods have been designed to identify multiple gene alterations simultaneously based on their co-occurrence or mutual exclusivity relationships in a tumor cohort, either with [13] or without [14, 15] reference to a molecular interaction network. Other approaches have been developed with the aim of identifying a specific subnetwork within a molecular interaction network, either through (i) a combinatorial formulation, with the goal of maximizing the *total weight* of the subnetwork in a molecular inter-

action network with node (and possibly edge) weights [16, 17], or (ii) a network diffusion process to derive specific mutated pathways [18, 19]. A direction particularly relevant to our paper is motivated by [20, 21, 18, 22], and explored by Bomersbach *et al.* [23], which proposed an alternative formulation for finding a subnetwork of a given size k with the goal of maximizing h, the number of samples for which at least one gene of the subnetwork is in an altered state. (A similar formulation where the goal is to maximize a weighted difference of h and k, for varying size k, can be found in [24].) Although the above combinatorial problems are typically NP-hard, they became manageable through the use of state of the art ILP (integer linear programming) solvers or greedy heuristics, or by the use of complex preprocessing procedures.

Complementary to the ideas proposed above, there are also several approaches to identify mutually exclusive (rather than jointly altered) sets of genes and pathways [25, 26, 27]. These approaches utilize the mutational heterogeneity prevalent in cancer genomes, and are driven by the observation that mutations acting on same pathway are many times mutually exclusive across tumor samples. Although, from a methodological point of view, these approaches are very interesting, they are not trivially extendable to the problem of identifying co-occurring alteration patterns (involving more than two genes) conserved across many samples.

1.1 Our Contributions

In this paper, we present a novel computational method, cd-CAP (**c**ombinatorial **d**etection of **C**onserved **A**lteration **P**atterns), which primarily incorporates an exhaustive search method (a variant of the a-priori algorithm) originally designed for association rule mining [28], to identify the largest subnetworks of an interaction network, each with an alteration pattern conserved across (a large subset of) a tumor sample cohort. cd-CAP exhibits significant speed advantage on available input datasets over the ILP formulations in [23, 24]. While the earlier attempt to overcome the performance barrier by ILP solvers relied on heuristics that can not guarantee optimality in solutions, our a-priori algorithm based approach does guarantee optimality while providing a similar running time performance available data sets. The key difference in the mathematical formulation of our method - which makes a data mining approach feasible (in contrast to [23, 24], which can not employ the a-priori algorithm) is that we ask each node in an identified subnetwork to have the same alteration type among the samples sharing it (although we have relaxed constraints that allow each sample to have a few nodes without any alterations). In addition, cd-CAP allows simultaneous identification of more than one subnetwork, and each gene within each subnetwork has labels specific to the alteration types it harbors. In fact, we allow a gene to have more than two labels (indicating whether it is mutated or not), each of which corresponds to a specific alteration type: somatic mutation, copy number alteration, or aberrant expression. From this point on we will refer to each distinct alteration type as a specific "color" of the corresponding node in an interaction network.

The algorithmic framework of cd-CAP consists of two major steps. The first step is the variant of a-priori algorithm to compute a set of all "candidate" subnetworks (each with a distinct color assignment) of size at most k shared among at least t samples (both k and t are user defined parameters). cd-CAP provides the user the additional options that (i) at least two distinct colors should be present in the coloring of a subnetwork, or (ii) each sample network can include up to a fraction δ of nodes whose color assignment differ from that of the "template". cd-CAP also gives the user option to stop at this point and provide (a) the largest colored subnetwork that appears in at least t samples (we report on some results obtained with this option), or (b) the colored subnetwork of size k that is shared by the largest number of samples. Alternatively, the second step solves the *maximum conserved subnetwork cover* problem which asks to cover the maximum

number of nodes in all samples with at most l colored subnetworks (l is user defined) - obtained in the first step - via ILP.

We have applied cd-CAP - with each of the possible options above, i.e., (i), (ii), (a) and (b) - to TCGA breast cancer (BRCA), colorectal adenocarcinoma (COAD), and glioblastoma (GBM) datasets, which collectively include over 1000 tumor samples. cd-CAP identified several connected subnetworks of interest, each exhibiting specific gene alteration pattern across a large subset of samples.

In particular, cd-CAP results with option (i) demonstrated that many of the largest highly conserved subnetworks within a tumor type solely consist of genes that have been subject to copy number gain, typically located on the same chromosomal arm and thus likely a result of a single, large scale amplification. One of these subnetworks cd-CAP observed (in about one third of the COAD samples [29]) include 9 genes in chromosomal arm 20q, which corresponds to a known amplification recurrent in colorectal tumors. Another copy-number gain subnetwork cd-CAP observed in breast cancer samples correspond to a recurrent large scale amplification in chromosome 1 [30]. It is interesting to note that cd-CAP was able to re-discover these events without specific training.

Several additional subnetworks identified by option (i) solely consist of genes that are aberrantly expressed. Further analysis with options (ii) and (b) of cd-CAP revealed subnetworks that capture signaling pathways and processes critical for oncogenesis in a large fraction of tumors. We have also demonstrated that the subnetworks identified through all three options of cd-CAP are associated with patients' survival outcome and hence are clinically important.

In order to assess the statistical significance of subnetworks discovered by cd-CAP - option (a), we introduce for the first time a model in which likely inter-dependent events, in particular amplification or deletion of all genes in a single chromosome arm, are considered as a single event. Conventional models of gene amplification either consider each gene amplification independently [31] (this is the model we implicitly assume in our combinatorial optimization formulations, giving a lower bound on the true p-value), or assumes each amplification can involve more than one gene (forming a subsequent sequence of genes) but with the added assumption that the original gene structure is not altered and the duplications occur in some orthogonal "dimension" [32, 33, 34]. Both models have their assumptions that do not hold in reality, but inferring evolutionary history of a genome with arbitrary duplications (that convert one string to another, longer string, by copying arbitrary substrings to arbitrary destinations) is NP-hard and even hard to approximate [35, 36]. By considering all copy number gain or loss events in the same chromosomal arm as a single event, we are, for the first time, able to compute an estimate that provides an empirical upper bound to the statistical significance (p-value) of the subnetworks discovered. (Note that this is not a true upper bound since a duplication event may involve both arms of a chromosome - but that would be very very rare.) Through this upper bound, together with the lower bound above, we can sandwich the true p-value and thus the significance of our discovery.

2 Methods

2.1 Combinatorial Optimization Formulation

Consider an undirected and node-labeled graph $G = (V, E)$, representing the human gene or protein interaction network, with n nodes where $v_j \in V$ represent genes and $e = (v_h, v_j) \in E$ represent interactions among the genes/proteins. Let us assume that we have m copies of the original network G, where each copy represents an individual sample P_i in a cohort. In each network $G_i = (V, E, C_i)$ corresponding to sample P_i, each node $v_{i,j}$ (as a copy of v_j) is colored with one or more possible colors to form the set $C_{i,j}$ (i.e. C_i maps $v_{i,j}$ to a possibly empty subset of colors $C_{i,j}$). Each color

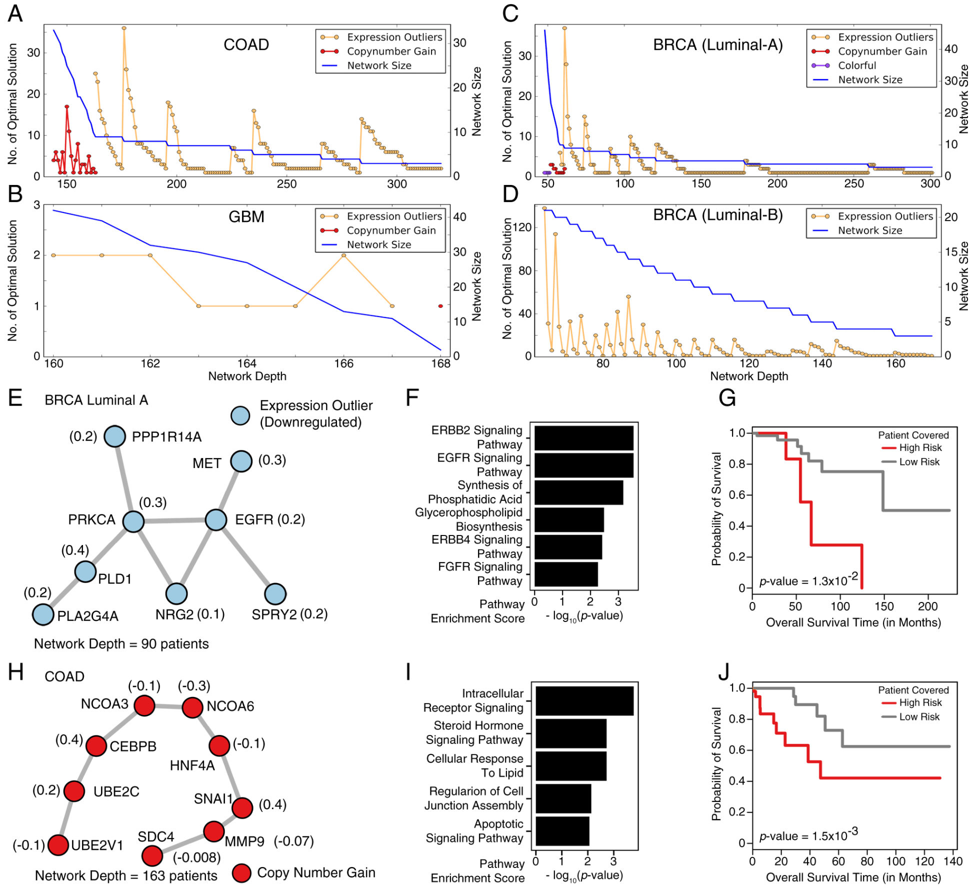

Figure 2: Conserved colored subnetworks. (A-D) Number of maximal solutions and the size of the conserved colored subnetwork obtained using the MCSI formulation, as a function of network depth t, in each of four cancer types analyzed, on STRING v10 (with high confidence edges) PPI network . The horizontal axis denotes the depth (number of patients) of the network. For the blue plot, the vertical axis denotes the maximum possible network size (in terms of the number of nodes) and thus it is strictly non-increasing by definition. For the plots with different colors, the vertical axis denotes the number of distinct networks with network size equal to that indicated by the blue plot. As can be seen, the red plots depict networks where all nodes have a copy number gain, the yellow plots depict networks where all nodes are expression outliers and purple plots depict colorful networks (with at least two distinct colors). A total of 41 subnetworks across all cancer types (10 COAD, 4 GBM, 11 Luminal A and 16 Luminal B) correspond to the end of valleys in the color plots - and were further analyzed. Two of the most interesting ones are provided here - both of which are singly colored. The number in parenthesis next to each node represents the univariate Cox proportional-hazard regression coefficient estimated for each gene, used as its weight in the risk-score calculation to stratify patients into two distinct risk groups. (See Methods section for details).
(E-G) One of the 11 maximal colored subnetworks identified in BRCA Luminal A dataset - it consists solely of downregulated expression outlier genes and has depth 90 (patients). (E) The colored subnetwork (with 8 nodes) topology. (F) Pathways dysregulated by alterations harboured by the genes in the subnetwork - these genes are involved in EGFR, ERBB2, and FGFR signaling pathways. (G) Kaplan-Meier plot showing the significant association of the subnetwork, with patients' clinical outcome. Patients "covered" by the subnetwork were stratified into two groups, namely High Risk (8 patients) vs Low Risk (82 patients), based on their gene expression levels. (See Methods for details.)
(H-J) One of the 10 maximal colored subnetworks identified in COAD dataset - it consists solely of copy number amplified genes and has depth of 163 (patients) . Genes in this subnetwork belong to the same chromosomal locus 20q13. (H) The colored subnetwork (with 9 nodes) topology. (I) Pathways dysregulated by the alterations harboured by the genes in the subnetwork - these genes are involved in signal transduction and apoptotic process. (J) Kaplan-Meier plot showing the significant association of the subnetwork with patients' clinical outcome (73 High Risk vs 83 Low Risk patients).

represents a distinct type of alteration harbored by a gene/protein, in particular somatic mutation (single nucleotide alteration or short indel), copy number gain, copy number loss or significant alteration in expression (which can be trivially expanded to include genic structural alteration - micro-inversion or duplication, gene fusion, alternative splicing, methylation altearation, non-coding sequence alteration) observed in the gene and the protein product. Without loss of generality, $C_{i,j} = \emptyset$ implies none of the possible alteration events are observed at $v_{i,j}$, and two nodes $v_{i,j}, v_{i',j}$ corresponding to each other in two distinct samples have at least one matching color if $C_{i,j} \cap C_{i',j} \neq \emptyset$.

The main goal of cd-CAP is to identify conserved patterns of (i.e. identically colored) connected subnetworks across a subset of sample networks G_i. Consider a connected subnetwork $T = (V_T, E_T)$ of the original interaction network G, where each node $v_j \in V_T$ is assigned exactly one color c_j. Such a colored subnetwork is said to be shared by a collection of sample networks $G_i (i \in I)$ if each node of the subnetwork harbor the same color in every sample network i.e. $c_j \in \bigcap_{i \in I} C_{i,j}$ for each $v_j \in V_T$. A colored node in a sample network is said to be covered by a subnetwork if the subnetwork is shared by the node's sample network (Figure 1). Intuitively, a colored subnetwork represents a conserved pattern or a network motif.

cd-CAP combinatorially formulates the problem of identifying conserved patterns of subnetworks as the **Maximum Conserved colored Subnetwork Identification problem (MCSI)**. Here the goal is to find the largest connected subnetwork S of the interaction network G, that occur in exactly t (a user specified number) samples $\mathcal{P}$, such that each node in S has the same color in each sample $P_i (\in \mathcal{P})$. Note that this formulation is orthogonal to that used in [23] and [24], where the goal is to maximize the number of samples that share a fixed size subnetwork. The advantage of formulating the problem as MCSI is that it naturally admits a generalization of the a-priori algorithm. We also note that our formulation considers distinct types of mutations (as colors) in the conserved alteration patterns, another key improvement to that used in [23, 24].

cd-CAP also supports simultaneous identification of multiple conserved subnetworks that are altered in a large number of samples. In one potential formulation of the problem one may aim to cover all nodes $v_{i,j}$ in all m input sample networks G_i, with the smallest number of subnetworks $T = (V_T, E_T)$ shared by at least one sample network. We refer this combinatorial optimization problem as **Minimum Subgraph Cover Problem for (Node) Colored Interaction Networks (MSC-NCI).**

One advantage of the MSC-NCI problem is that it is parameter-free. However, in a realistic multi-omics cancer dataset, the number of genes far exceeds the number of samples represented. Under such conditions, the solution to the MSC-NCI problem will primarily include subnetworks that are large connected components that are shared by only one sample network. To account for this situation, we introduce the following parameters/constraints akin to those for the MCSI formulation: (1) we require that the nodes in each subnetwork have the same color shared by at least t samples (in the remainder of the discussion, t is referred to as *depth* of a subnetwork); and (2) we require that each subnetwork returned contains at most k nodes. Note that this variant of the problem is infeasible for certain cohorts (consider a particular node which has a unique color for a particular sample; clearly requirement (1) can not be satisfied if $t > 1$). Even if there is a feasible solution, the requirement that each subnetwork in $\mathcal{T}$ is of size at most k makes the problem NP-hard (the reduction is from the problem of determining whether G can be exactly partitioned into connected subnetworks, each with k nodes [37]). As a result (3) we introduce one additional parameter, l, the maximum number of subnetworks (each of size at most k, and which are color-conserved in at least t samples) with the objective of covering the maximum number of nodes across all samples. We call the problem of identifying at most l subnetworks of size at most k, whose colors are conserved across at least t samples, so as to maximize the total number of nodes in all these samples covered by these subnetworks, as the **Maximum Conserved Subnetwork**

Coverage problem (MCSC).

2.2 Algorithmic Framework for solving MCSC

We formulate the MCSC problem (as well as MSC-NCI problem) as an ILP. A straightforward application of available ILP solvers can only handle relatively small instances of the MSC-NCI problem. This is because the number of variables and the number of constraints for the MSC-NCI ILP formulation are $O(n^2m^2)$ and $O(n^2m^3)$ respectively, both very large for a typical problem instance. Fortunately, in all instances of interest, only a limited number of genes are colored in comparison to the total number of nodes nm. This enables us to apply an exhaustive search method that is designed for association rule mining [28] to build a list of all candidate subnetworks *exactly and efficiently* (e.g. in comparison to the ILP or heuristic solutions in [23, 24]) and then solve the maximum coverage problem (MCSC) on the set of candidate subnetworks. Note that our exhaustive search method is an extension of the a-priori algorithm with the difference that we require the candidate subnetworks to maintain connectivity as they grow.

Generating Conserved Subnetworks. We generate the complete list of candidate subnetworks with minimum depth t by the use of "anti-monotone property" [38]: if any subnetwork S has depth $< t$, then the depth of all of its supergraphs $S' \supset S$ must be $< t$. This makes it possible to grow the set $\mathcal{S}$ of valid subnetworks comprehensively but without repetition (as described as "optimal order of enumeration" in [39]) through the following breadth-first network growth strategy.

(1) For every colored node $v_{i,j}$ and each of its colors c_ℓ, we create a candidate subnetwork of size 1 containing the node with color c_ℓ. All samples in which the node is colored c_ℓ naturally share this trivial subnetwork.

(2) We inductively consider all candidate subnetworks of size s with the goal of growing them to subnetworks of size $s+1$ as follows. For a given subnetwork T of size s, consider each neighboring node u. For each possible color c'_ℓ of u, we create a new candidate subnetwork of size $s+1$ by extending T with u - with color c'_ℓ. We maintain this subnetwork for the next inductive step only if the number of samples sharing this new subnetwork is at least t; otherwise, we discard it.

During the extension of T above, if the new node u does not reduce the number of samples sharing it, T becomes redundant and is not considered in the ILP formulation.

Solving MCSC. Given the universe $\mathcal{U} = \{v_{i,j} \,|\, C_{i,j} \neq \emptyset\,, i = 1, \cdots, m; j = 1, \cdots, n\}$, containing all the coloured nodes in all the sample networks, and the collection of all subnetworks $\mathcal{S} = \{T_i \,|\, T_i \text{ is shared by at least } t \text{ samples and contains at most } k \text{ nodes}\}$, our goal is to identify up to l subnetworks from the collection $\mathcal{S}$ whose union contains the maximum possible number of elements of the universe $\mathcal{U}$.

$$\text{Maximize} \quad \sum_{v_{i,j} \in \mathcal{U}} C[i,j]$$

$$\text{s.t.} \quad \sum_{T_p \in \mathcal{S}_{i,j}} X[p] \geq C[i,j] \quad (\forall v_{i,j} \in \mathcal{U})$$

$$\sum_{T_i \in \mathcal{S}} X[i] \leq l$$

After the list of all candidate subnetworks $\mathcal{S}$ is constructed (as described in the previous subsec-

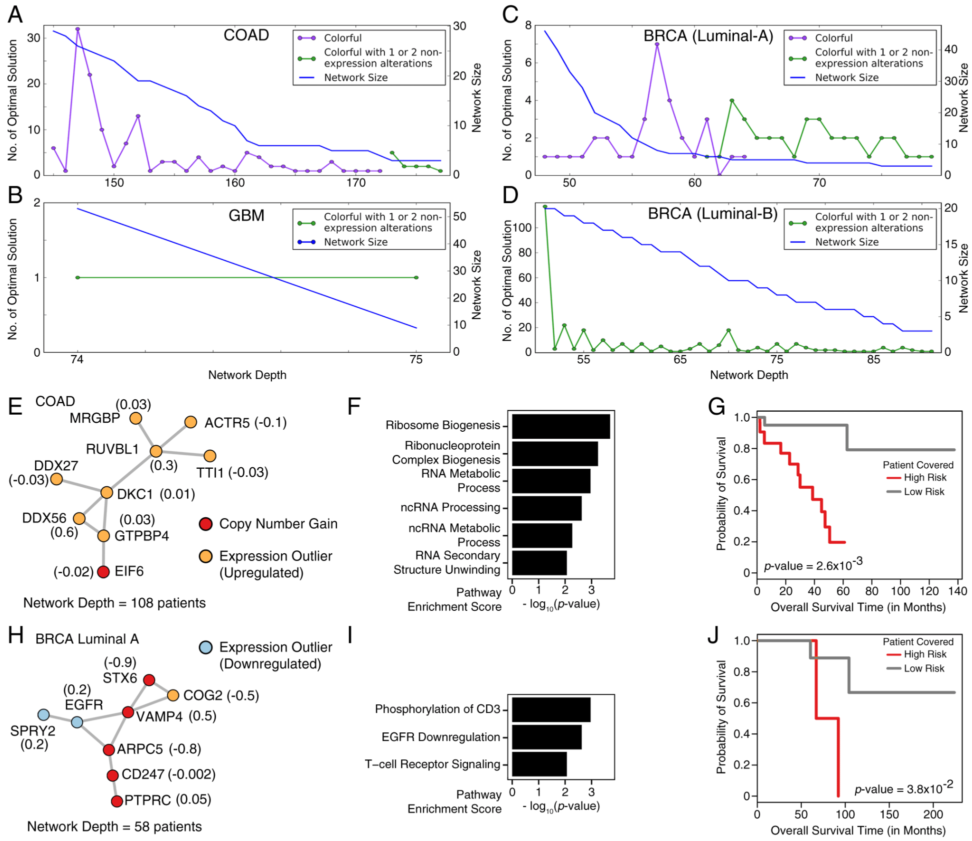

Figure 3: Colorful maximal subnetworks.
(A-D) Number of maximal solutions and the size of the conserved colorful subnetwork obtained using the MCSI formulation, as a function of network depth t, in each of four cancer types analyzed on the STRING v10 (high confidence edges) PPI network. The horizontal axis denotes the depth (number of patients) of the network. For the blue plot, the vertical axis denotes the maximum possible network size (in terms of the number of nodes) and thus it is strictly non-increasing by definition. For the plots with different colors, the vertical axis denotes the number of distinct networks with network size equal to that indicated by the blue plot. As can be seen, the purple plots depict colorful subnetworks and the green plots depict networks that include one to two nodes which are not expression outliers. A similar analysis was performed on the STRING v10 (experimentally validated edges), REACTOME and HPRD PPI networks. A total of 104 colorful subnetworks corresponding to the end of valleys of the plots were identified across the 4 cancer types in all the above PPI networks. Two of the most interesting ones are provided here. The number in parenthesis next to each node represents the univariate Cox proportional-hazard regression coefficient estimated for that gene, used as its weight in the risk-score calculation to stratify the patients into two distinct risk groups. (See Methods section for details).
(E-G) One of the maximal colorful subnetworks identified in the COAD dataset, consisting of at most 2 non-expression outlier (for this case copynumber gain) genes, with depth 108 (patients). (E) The colored subnetwork (with 9 nodes) topology - obtained from STRING v10 (with experimentally validated edges) PPI network. (F) Pathways dysregulated by alterations harboured by the genes in the subnetwork - these genes are involved in Ribosome biogenesis and RNA processing. (G) Kaplan-Meier plot showing the significant association of the subnetwork, with patients' clinical outcome (59 High Risk vs 47 Low Risk patients).
(H-J) One of the maximal colorful subnetworks identified in the Luminal A dataset with no color restrictions, with depth of 58 (patients). (H) The colored subnetwork (with 8 nodes) topology - obtained in the REACTOME PPI network. (I) Pathways dysregulated by the alterations harboured by the genes in the subnetwork. (J) Kaplan-Meier plot showing the significant association of the subnetwork with patients' clinical outcome (30 High Risk vs 30 Low Risk patients).

tion), we represent the MCSC problem with the following integer linear program and solve it using IBM ILOG CPLEX or Gurobi. A binary variable $C[i,j]$ corresponds to whether colored node $v_{i,j}$ was covered by at least one chosen subnetwork, and binary variable $X[i]$ corresponds to whether colored candidate subnetwork T_i was one of the chosen. Let $\mathcal{S}_{i,j}$ represent the set of all subnetworks of $\mathcal{S}$ which contain node $v_{i,j}$ properly colored in them.

Special Types of Conserved Subnetworks. In addition to the exactly-conserved colored subnetworks obtained through the general MCSC formulation, we also consider two important variants. (1) *Colorful Conserved Subnetworks.* A colorful subnetwork T is one that has at least two distinct colors represented in the coloring of its nodes, i.e. $c_\ell, c_h \in \bigcap_{v_j \in T} C_j \quad (c_\ell \neq c_h)$. In some of the datasets that we analyzed, certain colors were dominant in the input to such extent that all subnetworks identified by our method had all nodes colored the same. By restricting focus to *colorful* subnetworks, it is possible, e.g., to capture conserved patterns of potential driver alterations and their impact on their vicinity in the interaction network, in the form of expression alterations. In order to identify the maximalcolorful conserved subnetwork of a given depth t in the tumor samples, we only need to keep track of the colorful subnetworks in each iteration - since any colorful network must contain a connected colorful subnetwork.
(2) *Subnetworks Conserved within error rate* δ. In order to reduce the sensitivity of our method to noise (or lack of precision in generating the data) in the input when detecting conserved patterns, we extend our formulation to allow some "errors" in identifying conserved subnetworks. We define δ, the error rate of a colored subnetwork T as the maximum allowable fraction of nodes of T without an assigned color in any sample P_i that shares T. For tolerating an error rate of δ, we extend our algorithm to generate candidate subnetworks $\mathcal{S}$ for the MCSC problem by performing a post-processing step in which the list of samples sharing subnetwork T is increased by including all samples that share T with an error rate of δ. (Note that our notion of error is restricted to nodes that do not have a color, i.e. an observed alteration, in each specific sample.)

3 Network Analysis

3.1 Significance of the Identified Subnetworks

Under the assumption that each gene is altered independently, it is possible to apply the conventional permutation test [27, 13, 23], to assess the statistical significance of the subnetworks identified by cd-CAP as follows. Let $\mathcal{C}_i = \{(v_{i,j}, c) : c \in C_{i,j} \neq \emptyset, v_{i,j} \in V\}$ be a binary relation representing the existing colors on each node of sample network G_i. A permuted copy of the interaction network $G'_i = (V, E, C'_i)$ is generated (under the null hypothesis) by randomly shuffling the range of $\mathcal{C}$, such that each node $v_{i,j}$ takes a new set of colors $C'_{i,j}$ with the total number of colors $\sum_j |C_{i,j}|$ in G_i preserved. (In other words, $\sum_j |C_{i,j}| = \sum_j |C'_{i,j}|$, and a simple implementation assigns $|C'_{i,j}|$ by random shuffling $(|C_{i,j}| : j = 1, 2, \cdots, n)$.) An entire set of permuted sample networks consists of each randomly generated G'_i, and this permutation test is repeated sufficiently many (by default 1000) times. For a particular size k subnetwork $T = (V_T, E_T)$ identified by cd-CAP (on t samples) we define P_1 as the fraction of these permutation tests where *any* subnetwork of size at least k appear in t or more samples.

In fact, P_1 presents a lower bound on the p-value for T since it ignores the inter-dependency of node colors (gene alteration events). In particular, whole chromosome or chromosome arm level copy number amplifications/deletions are commonly observed in various cancer types. To address this issue, we apply the following procedure to calculate P_2 as an empirical upper-bound for the p-value of T, under the assumption that copy number alterations take place in whole chromosome

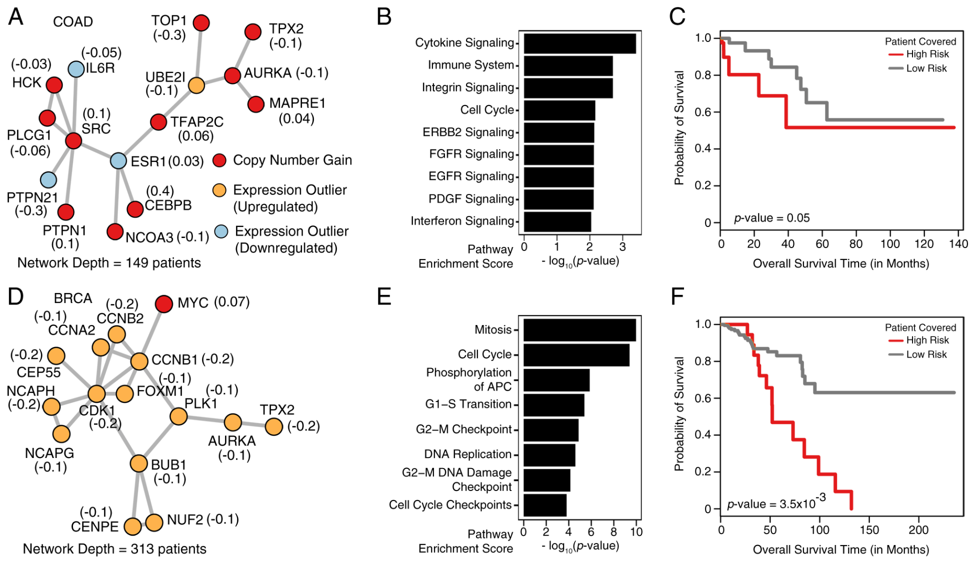

Figure 4: Multiple Subnetwork Analysis. Two largest among the 15 subnetworks identified across the COAD, GBM and BRCA data sets (5 per each) through the MCSC formulation of cd-CAP on STRING v10.5 (with experimentally validated edges) PPI network. The number in parenthesis next to each node represents the univariate Cox proportional-hazard regression coefficient estimated for that gene, used as its weight in the risk-score calculation to stratify the patients into two distinct risk groups. (See Methods section for details).
(A-C) The largest of the 5 COAD subnetworks with a network depth of 149 (patients). (A) The subnetwork topology (with 15 nodes).
(B) Pathways dysregulated by alterations harboured by the genes in the subnetwork. (C) Kaplan-Meier plot showing the significant association of the subnetwork, with patients' clinical outcome (69 High Risk vs 78 Low Risk patients).
(D-F) The largest of the 5 BRCA subnetworks with a network depth of 313 (patients). (D) The subnetwork topology (with 15 nodes). (E) Pathways dysregulated by the alterations harboured by the genes in the subnetwork. (F) Kaplan-Meier plot showing the significant association of the subnetwork with patients' clinical outcome (33 High Risk vs 278 Low Risk patients).

arms. First we identify all genes $v_j \in V$ on the same chromosome arm, $\mathrm{chr}(v_j)$ and construct a set of supernodes $U_i^{\mathrm{chr}} = \{\mathrm{chr}(v_{i,j}) : \exists c,\ (v_{i,j}, c) \in \mathcal{C}_i\}$ from the genes on the same chromosome arm for each sample P_i. Let $N_E = |\{(v_{i,j}, E) \in \mathcal{C}_i\}|$ denote the number of nodes with color E (corresponding to either a copy number gain or loss) in sample P_i. Then, each supernode is assigned the color E independently with probability $\frac{N_E}{|\mathcal{C}_i|}$, which guarantees that the expected count of E in P_i is preserved. Finally we randomly assign the remaining colors to those nodes without a color assignment thus far, to obtain a new randomly permuted interaction network $G_i'' = (V, E, C_i'')$ towards an empirical p-value (upper bound) estimate. We again repeat this process sufficiently many (by default 1000) times to generate distinct permuted datasets and derive P_2 by counting the fraction of these datasets where any subnetwork of size at least k appear in t or more samples. The true statistical significance is expected to be in the range $[P_1, P_2]$ provided that chromosome arms form the largest units of alteration.

3.2 Pathway enrichment analysis

The set of genes in the subnetwork were tested for enrichment against gene sets of pathways present in the Molecular Signature Database (MSigDB) v6.0 [40]. A hypergeometric test based gene set enrichment analysis [40] was used for this purpose. A cut-off threshold of false discovery rate (FDR) ≤ 0.01 was used to obtain the significantly enriched pathways.

3.3 Association of sub-networks with patients' survival outcome

In order to assess the association of identified subnetworks with patients' survival outcome, we used a risk-score based on the (weighted) aggregate expression of the genes in the subnetwork. The risk-score (S) of a patient is defined as the sum of the normalized gene-expression values in the subnetwork, each weighted by the estimated univariate Cox proportional-hazard regression coefficient [41], i.e., $S = \sum_i^k \beta_i x_{ij}$. Here i and j represents a gene and a patient respectively, β_i is the coefficient of Cox regression for gene i, x_{ij} is the normalized gene-expression of gene i in patient j, and k is the number of genes in the subnetwork. The normalized gene-expression values were fitted against overall survival time with living status as the censored event using univariate Cox proportional-hazard regression (exact method). Based on the risk-score values, patients were stratified into two groups: low-risk group (patients with $S <$ mean of S), and high-risk group (patients with $S \geq$ mean of S). Note that only those patients that are covered by the subnetwork are considered for the analysis above.[1]

4 Results

We obtained somatic mutation, copy number aberration and RNA-seq based gene-expression data from three distinct cancer types - glioblastoma multiforme (GBM) [42], breast adenocarcinoma (BRCA) [43], and colon adenocarcinoma (COAD) [29] from The Cancer Genome Atlas (TCGA) datasets (the detailed information can be found in Supplementary Section 1). In addition, we distinguish four commonly observed molecular subtypes (i.e. Luminal A, Luminal B, Triple-negative/basal-like and HER2-enriched) from the BRCA cohort. For each sample, we obtained

[1] In fact, the set of patients covered by a subnetwork identified by cd-CAP would not necessarily differ from those patients without the subnetwork with respect to survival outcomes, because the latter set is likely composed of highly heterogeneous cancer subtypes.

the list of genes which harbor somatic mutations, copy number aberrations, or are expression outliers as per below.

Somatic Mutations. All non-silent variant calls that were identified by at least one tool among MUSE, MuTect2, SomaticSniper and VarScan2 were considered.

Copy Number Aberrations. CNA segmented data from NCI-GDC were further processed using Nexus Copy Number Discovery Edition Version 9.0 (BioDiscovery, Inc., El Segundo, CA) to identify aberrant regions in the genome. We restricted our analysis to the most confident CNA calls selecting only those genes with high copy gain or homozygous copy loss.

Expression outliers. We used HTSeq-FPKM-UQ normalized RNA-seq expression data to which we applied the generalized extreme studentized deviate (GESD) test [44]. In particular, we used GESD test to compare the transcriptome profile of each tumor sample (one at a time) with that from a number of available normal samples. For each gene, if the tumor sample was identified as the most extremely deviated sample (using critical value $\alpha = 0.1$), the corresponding gene was marked as an expression-outlier for that tumor sample. This procedure was repeated for every tumor sample. Finally, comparing the tumor expression profile of these outlier genes to the normal samples, their up or down regulation expression patterns were determined.

4.1 Maximal Colored Subnetworks Across Cancer Types

We used cd-CAP to solve the maximum conserved colored subnetwork identification problem exactly in (each one of the four) protein-interaction network(s) on each cancer type - for every feasible value of network depth. As can be easily observed, the depth and the size of the identified subnetwork are inversely related. We say that a given value of the network depth is feasible if (i) the depth is at least 10% of the cohort size, (ii) the maximum network size for that depth is at least 3, (iii) the number of "candidate" subnetworks are at most 2 millions per iteration when running cd-CAP for that depth.

The number of maximal solutions of cd-CAP as a function of feasible network depth for each cancer type (COAD, GBM, BRCA Luminal A, and BRCA Luminal B) is shown in Figure 2A-D on STRING v10 PPI network with high confidence edges (see Supplementary Figure 2-5 for the results on alternative PPI networks). In general, for a fixed network size, the number of distinct networks of that size decreases as the network depth increases. One can observe that the end of "valleys" in the colored plots in Figure 2A-D correspond to the largest depth that can be obtained for a given subnetwork size. Throughout the remainder of the paper we focus on the colored subnetworks of each given size making the network depth maximum possible - which correspond to the end of valleys in the plots. If for a given subnetwork size and the corresponding maximal depth, cd-CAP returns more than 1 subnetwork, we discard those solutions.

Most of the subnetworks, especially those with large depth, identified for each of the four cancer types consisted of expression outlier genes (typically all upregulated or all downregulated) only (Figure 2A-D)[2]. As the network depth decreases, maximal subnetworks that consist only of copy number variants emerge. One of the most prominent copy-number gain subnetworks of the COAD dataset has depth 163 out of 463 patients in the cohort. This network forms the core of the larger maximal subnetworks cd-CAP identifies for lower depth values; it corresponds to a copy

[2]Note that several subnetworks identified in this analysis turned out to be uni-colored. A comparison of cd-CAP's performance with a limited version of cd-CAP that does not differentiate mutation types with respect to survival outcomes in TCGA COAD, GBM and BRCA data sets are provided in Supplementary Figure 1. cd-CAP's ability to distinguish mutation types in the networks shared across patients are visible in COAD and GBM data sets. In the BRCA data set, since all subnetworks are interest involve differentially expressed genes, the survival outcome differences are much less significant.

number gain of the chromosomal arm 20q - a known copy number aberration pattern highly specific to colorectal adenocarcinoma tumors [29].

Another subnetwork cd-CAP identified in 15% of the 422 BRCA Luminal-A samples corresponds to a copy number gain on chromosome 1 which is again a known aberration associated with breast cancer [30]. With increasing depth, the maximal subnetworks cd-CAP identifies in Luminal A cohort start to consist solely of expression outlier genes. In particular cd-CAP identified a subnetwork of eight underexpressed genes with network depth 90 (Figure 2E) - consisting of genes *EGFR*, *PRKCA*, *SPRY2*, and *NRG2*, known to be involved in EGFR/ERBB2/ERBB4 signaling pathways (Figure 2F). *EGFR* is an important driver gene involved in progression of breast tumors to advanced forms [45] and its altered expression is observed in a number of breast cancer cases [30]. The subnetwork also included *MET*, another well-known oncogene [46], and is enriched for members of the Ras signaling pathway, which is also known for its role in oncogenesis and mediating cancer phenotypes such as over-proliferation [47].

Next, for each patient covered by the subnetwork, we calculated a risk-score defined as a linear combination of the normalized gene-expression values of the genes in the subnetwork weighted by their estimated univariate Cox proportional-hazard regression coefficients (see Methods section for details). Based on the risk-score values, the patients covered by the subnetwork were stratified into two risk groups. Luminal A subnetwork was the most significant among all subnetworks identified in this dataset (Figure 2G). The patients in the high-risk group have poor overall survival outcome suggesting clinical importance of the identified subnetwork by cd-CAP.

As another example, we identified a colored subnetwork with copy number gain genes that covered 163 patients in the COAD dataset (Figure 2H). The genes in this subnetwork belong to the same chromosome locus 20q13, suggesting that they may comprise a single region of chromosomal amplification. Intriguingly all the members forms a linear pathway-like structure also on the PPI level. Among them is a group of functionally related genes consisting of transcription factors and their regulators (genes *CEBPB*, *NCOA's*, *UBE2's*), which are known to be involved in the intracellular receptor signaling pathway (Figure 2I). *CEBPB* and *UBE2's* are also involved in the regulation of cell cycle [48]. To the other end of the linear subnetwork, we found *MMP9* and *SDC4*, the established mediators of cancer invasion and apoptosis [49, 50]. Also we confirmed that this set of genes are highly predictive of the patients' survival outcome (Figure 2J). These results support the functional importance and clinical relevance of the subnetwork we identified.

4.2 Maximal Colorful Subnetworks Across Cancer Types

We next used cd-CAP to solve the maximum conserved colored subnetwork identification problem - with at least two distinct colors (see Section 2.2 for details), in each of the four protein-interaction network(s) and on each cancer type. Again, cd-CAP was run with every feasible value (as defined above) of network depth. The number of maximal solutions of cd-CAP as a function of network depth for each cancer type (COAD, GBM, BRCA Luminal A, and BRCA Luminal B) is shown in Figure 3A-D on STRING v10 PPI network with high confidence edges (see Supplementary Figure 2-4 for the results on alternative PPI networks). Note that we distinguish here the maximal subnetworks with one or two sequence-level alterations (i.e. somatic mutations and copy number alterations) – which is of potential interest since their neighboring expression-level alterations are possibly caused by these sequence-level alterations (Figure 3E provides an example) – with all the other cases. Similarly, we only focus on the maximal colorful subnetworks of every possible size for which the network depth is maximum possible and discard the solutions when cd-CAP returns more than 1 colorful subnetworks for each feasible value of network depth.

One colorful COAD subnetwork of note is composed of overexpressed genes with an additional

copy number gain gene that covers 108 patients (Figure 3E). This subnetwork is mainly enriched for genes involved in ribosome biogenesis (Figure 3G). Cancer has been long known to have an increased demand on ribosome biogenesis [51], and increased ribosome generation has been reported to contribute to cancer development [52]. The biological relevance of this subnetwork is also supported by survival analysis, which shows a strong differentiation between the high-risk and low-risk groups - see Figure 3F.

Another colorful subnetwork we observed in 58 BRCA Luminal A samples consists of four copy number gained genes, an overexpressed gene, and two underexpressed genes, including *EGFR* (Figure 3H). All copy-number gained genes and the overexpressed gene are located in chromosome 1q, commonly reported in breast cancer [30]. The subnetwork involves an interesting combination of the down-regulation of the cancer gene *EGFR* and the amplification of a group of genes involved in T-cell receptor signaling (*PTPRC*, *CD247*, and *ARPC5*; see Figure 3I). Thus we may surmise that the covered population of patients potentially have relatively low cancer proliferation index with higher anti-tumor immune response, which can be highly relevant indicators with regard to clinical outcome. Indeed, this subnetwork is significantly associated with patients' survival (Figure 3J).

4.3 Multiple-Subnetwork Analysis Across Cancer Types

We next sought to detect up to 5 subnetworks per cancer type that collectively cover maximum possible number of colored nodes by solving the MCSC problem on STRING v10.5 network (with experimentally validated edges). The subnetwork extension error rate was set to 20%, and we restricted the search space to subnetworks which do not consist only of expression outlier nodes, in order to obtain what we believe to be more biologically interesting results. Parameter t was chosen for each dataset in a way that made it possible to construct all candidate subnetworks of maximum possible size while keeping the total number of candidate subnetworks below 2×10^6, making the problem solvable in reasonable amount of time. We set t to 69 (15% of the patients), 62 (10% of the patients), and 110 (10% of the patients) respectively for COAD, GBM, and BRCA datasets. Supplementary Table 1 shows the size, per sample depth and the coloring of the nodes in the resulting subnetworks.

We note that the subnetworks identified in the GBM dataset had the lowest depth (10-15% of the samples). COAD and BRCA datasets on the other hand have much larger depth (respectively 30-48% and 15-32% of the samples). Smaller subnetworks of the GBM dataset solely consist of copy number gain genes on chromosome 7q, a known amplification in GBM [53]. The two large subnetworks each contain a single gene with copy number gain (*SEC61G* and *EGFR*, respectively) accompanied by several of overexpressed genes. BRCA dataset exhibits a similar pattern: each of the four large subnetworks contain a single copy number gain gene from chromosome 8q, (*NSMCE2* in one and *MYC* in the remaining three subnetworks). Subnetworks detected in COAD dataset were much more colorful and recurrently conserved in a larger fraction of samples than those in the other datasets. All genes with copy number gain are located in chromosome 20q.

We identified a subnetwork with 15 nodes (11 genes with copy number gain, 1 overexpressed and 3 underexpressed genes) in 149 COAD patients (Figure 4A). All 11 copy number gain genes belong to chromosome 1q. *IL6R*, *PLCG1*, *PTPN1*, and *HCK* are involved in cytokine/interferon signaling to activate immune cells to counter proliferating tumor cells [54] (Figure 4B). *UBE2I*, *AURKA*, and *MAPRE1* are involved in cell cycle processes. This subnetwork was found to be associated with patients' survival outcome (Figure 4C).

We identified another subnetwork with 15 nodes (14 overexpressed and 1 copy number gain genes) in 313 breast cancer patients (Figure 4D). Genes in this subnetwork are involved in cell

cycle processes (Figure 4E). In particular the cell cycle checkpoint processes were dysregulated - which is known to drive tumor initiation processes [55]. The subnetwork was found to be associated with patients' survival outcome (Figure 4F) demonstrating its clinical relevance.

4.4 Empirical P-Value Estimates Confirm the Significance of cd-CAP Identified Networks

To evaluate the significance of cd-CAP's findings, we performed the permutation test in Section 2.3 1000 times on each cancer type for each setting of subnetwork constraints. Supplementary Table 2-3 and Figure 6 demonstrate the distribution of the empirical p-value upper bound estimates with STRING 10 (high confidence edges) PPI network, while the lower bound results look similar to what is presented in the figure and thus are omitted. In the permutation tests all cd-CAP identified subnetworks (without additional constraints) of size 2-5 were composed solely of expression altered genes; in contrast there are several larger CNV rich subnetworks observed in the TCGA COAD data set and others, further confirming the significance of our findings. Colorful subnetworks presented in Figure 3 are even less likely to occur at random (we therefore omit empirical p-value estimates for the networks in Figure 3).

5 Discussion

In this paper we introduce a novel combinatorial framework and an associated tool named cd-CAP which can identify (one or more) subnetworks of an interaction network where genes exhibit conserved alteration patterns across many tumor samples. Compared with the state-of-the-art methods (e.g.[22, 24]), cd-CAP differentiates alteration types associated with each gene (rather than relying on binary information of a gene being altered or not), and simultaneously detects multiple *alteration type conserved* subnetworks.

cd-CAP provides the user with two major options. (a) It computes the largest colored subnetwork that appears in at least t samples. This option exhibits significant speed advantage over available ILP-based approaches; its a-priori based algorithmic formulation allows flexible integration of special constraints (on maximal subnetworks) – not only simplifying complicated ILP constraints, but also further reducing the number of candidate subnetworks in iteration steps (a good example for this is the "colorful conserved subnetworks" as introduced in Section 2.2). However, the identified subnetworks are required to be *conserved*, i.e., each node only admits one alteration type among the samples sharing it (although we have relaxed constraints that allow each sample to have a few nodes without any alterations, i.e. colors). In the future, we may extend the definition of a network to include nodes with color mismatches (for example, according to the definition in [22] or [21]) with a modification to cd-CAP's candidate subnetwork generation algorithm. (b) It solves the *maximum conserved subnetwork cover* (MCSC) problem to cover the maximum number of nodes in all samples with at most l colored subnetworks (l is user defined) via ILP. In the future we aim to refine the MCSC formulation with reduced number of parameters and hope to develop exact or approximate solutions.

Subnetworks identified by cd-CAP in COAD, GBM and BRCA datasets from TCGA are typically enriched with genes harboring gene-expression alterations or copy-number gain. Notably, we observed that genes in subnetworks with copy-number amplification are universally located in the same chromosomal locus. Many of these genes have known interactions and are functionally similar, demonstrating the ability of cd-CAP in capturing functionally active subnetworks, conserved across a large number of tumor samples. These subnetworks seem to overlap with pathways critical for oncogenesis. In the datasets analyzed, we observed cell cycle, apoptosis, RNA processing,

and immune system processes that are known to be dysregulated in a large fraction of tumors. cd-CAP also captured subnetworks relevant to EGFR/ERBB2 signaling pathways, which have distinct expression patterns in specific subtypes of breast cancer [56, 30]. Survival analysis of cd-CAP identified subnetworks also confirmed their substantial clinical relevance.

Software and Code Availability

cd-CAP is implemented in C++. The source is available through: `https://github.com/ehodzic/cd-CAP`.

References

[1] Vogelstein, B. *et al.* Cancer genome landscapes. *Science (New York, N.Y.)* **339**, 1546–58 (2013).

[2] Shrestha, R. *et al.* HIT'nDRIVE: patient-specific multidriver gene prioritization for precision oncology. *Genome research* **27**, 1573–1588 (2017).

[3] Bailey, M. H. *et al.* Comprehensive Characterization of Cancer Driver Genes and Mutations. *Cell* **173**, 371–385.e18 (2018).

[4] Alexandrov, L. B. *et al.* Signatures of mutational processes in human cancer. *Nature* **500**, 415–21 (2013).

[5] Carver, B. S. *et al.* Aberrant ERG expression cooperates with loss of PTEN to promote cancer progression in the prostate. *Nature Genetics* **41**, 619–624 (2009).

[6] King, J. C. *et al.* Cooperativity of TMPRSS2-ERG with PI3-kinase pathway activation in prostate oncogenesis. *Nature Genetics* **41**, 524–526 (2009).

[7] Kron, K. J. *et al.* TMPRSS2-ERG fusion co-opts master transcription factors and activates NOTCH signaling in primary prostate cancer. *Nature Genetics* **49**, 1336–1345 (2017).

[8] Chen, F. *et al.* Multilevel Genomics-Based Taxonomy of Renal Cell Carcinoma. *Cell Reports* **14**, 2476–2489 (2016).

[9] Robertson, A. *et al.* Integrative Analysis Identifies Four Molecular and Clinical Subsets in Uveal Melanoma. *Cancer Cell* **32**, 204–220 (2017).

[10] Yoshikawa, Y. *et al.* High-density array-CGH with targeted NGS unmask multiple noncontiguous minute deletions on chromosome 3p21 in mesothelioma. *Proceedings of the National Academy of Sciences of the United States of America* **113**, 13432–13437 (2016).

[11] Campbell, P. J. Cliques and Schisms of Cancer Genes. *Cancer Cell* **32**, 129–130 (2017).

[12] Campbell, P. J. *et al.* Pan-cancer analysis of whole genomes. *bioRxiv* (2017).

[13] Kim, Y.-A., Cho, D.-Y., Dao, P. & Przytycka, T. M. Memcover: integrated analysis of mutual exclusivity and functional network reveals dysregulated pathways across multiple cancer types. *Bioinformatics* **31**, i284–i292 (2015).

[14] Mina, M. *et al.* Conditional Selection of Genomic Alterations Dictates Cancer Evolution and Oncogenic Dependencies. *Cancer cell* **29**, 723–736 (2017).

[15] Dao, P. *et al.* BeWith: A Between-Within method to discover relationships between cancer modules via integrated analysis of mutual exclusivity, co-occurrence and functional interactions. *PLoS computational biology* **13**, e1005695 (2017).

[16] El-Kebir, M. & Klau, G. W. Solving the maximum-weight connected subgraph problem to optimality. *CoRR* **abs/1409.5308** (2014).

[17] Loboda, A. A., Artyomov, M. N. & Sergushichev, A. A. Solving generalized maximum-weight connected subgraph problem for network enrichment analysis. In *International Workshop on Algorithms in Bioinformatics*, 210–221 (Springer, 2016).

[18] Vandin, F., Upfal, E. & Raphael, B. J. Algorithms for detecting significantly mutated pathways in cancer. *Journal of Computational Biology* **18**, 507–522 (2011).

[19] Leiserson, M. D. M. *et al.* Pan-cancer network analysis identifies combinations of rare somatic mutations across pathways and protein complexes. *Nature Genetics* **47**, 106–114 (2014).

[20] Kim, Y.-A., Salari, R., Wuchty, S. & Przytycka, T. M. Module cover–a new approach to genotype-phenotype studies. In *Pacific Symposium on Biocomputing. Pacific Symposium on Biocomputing*, 135 (NIH Public Access, 2013).

[21] Ulitsky, I., Krishnamurthy, A., Karp, R. M. & Shamir, R. DEGAS: de novo discovery of dysregulated pathways in human diseases. *PloS one* **5**, e13367 (2010).

[22] Alcaraz, N. *et al.* Efficient key pathway mining: combining networks and omics data. *Integrative Biology* **4**, 756–764 (2012).

[23] Bomersbach, A., Chiarandini, M. & Vandin, F. An efficient branch and cut algorithm to find frequently mutated subnetworks in cancer. In *International Workshop on Algorithms in Bioinformatics*, 27–39 (Springer, 2016).

[24] Hristov, B. H. & Singh, M. Network-based coverage of mutational profiles reveals cancer genes. *Cell Systems* **5**, 221–229.e4 (2017).

[25] Miller, C. A., Settle, S. H., Sulman, E. P., Aldape, K. D. & Milosavljevic, A. Discovering functional modules by identifying recurrent and mutually exclusive mutational patterns in tumors. *BMC medical genomics* **4**, 34 (2011).

[26] Ciriello, G., Cerami, E., Sander, C. & Schultz, N. Mutual exclusivity analysis identifies oncogenic network modules. *Genome research* **22**, 398–406 (2012).

[27] Vandin, F., Upfal, E. & Raphael, B. J. De novo discovery of mutated driver pathways in cancer. *Genome research* **22**, 375–85 (2012).

[28] Agrawal, R. & Srikant, R. Fast algorithms for mining association rules in large databases. In *Proceedings of the 20th International Conference on Very Large Data Bases*, VLDB '94, 487–499 (Morgan Kaufmann Publishers Inc., San Francisco, CA, USA, 1994).

[29] Cancer Genome Atlas Network. Comprehensive molecular characterization of human colon and rectal cancer. *Nature* **487**, 330–7 (2012).

[30] Curtis, C. *et al.* The genomic and transcriptomic architecture of 2,000 breast tumours reveals novel subgroups. *Nature* **486**, 346–52 (2012).

[31] Chowdhury, S. A. *et al.* Algorithms to model single gene, single chromosome, and whole genome copy number changes jointly in tumor phylogenetics. *PLoS computational biology* **10**, e1003740 (2014).

[32] Schwarz, R. F. *et al.* Phylogenetic quantification of intra-tumour heterogeneity. *PLoS computational biology* **10**, e1003535 (2014).

[33] El-Kebir, M. *et al.* Copy-number evolution problems: complexity and algorithms. In *International Workshop on Algorithms in Bioinformatics*, 137–149 (Springer, 2016).

[34] Zaccaria, S., El-Kebir, M., Klau, G. W. & Raphael, B. J. The copy-number tree mixture deconvolution problem and applications to multi-sample bulk sequencing tumor data. In *International Conference on Research in Computational Molecular Biology*, 318–335 (Springer, 2017).

[35] Cormode, G., Paterson, M., Sahinalp, S. C. & Vishkin, U. Communication complexity of document exchange. In *Proceedings of the eleventh annual ACM-SIAM symposium on Discrete algorithms*, 197–206 (Society for Industrial and Applied Mathematics, 2000).

[36] Muthukrishnan, S. & Sahinalp, S. C. Approximate nearest neighbors and sequence comparison with block operations. In *Proceedings of the thirty-second annual ACM symposium on Theory of computing*, 416–424 (ACM, 2000).

[37] Dyer, M. E. & Frieze, A. M. On the complexity of partitioning graphs into connected subgraphs. *Discrete Applied Mathematics* **10**, 139–153 (1985).

[38] Leung, C. K.-S. Anti-monotone constraints. In *Encyclopedia of Database Systems*, 98–98 (Springer, 2009).

[39] Maxwell, S., Chance, M. R. & Koyutürk, M. Efficiently enumerating all connected induced subgraphs of a large molecular network. In *International Conference on Algorithms for Computational Biology*, 171–182 (Springer, 2014).

[40] Subramanian, A. *et al.* Gene set enrichment analysis: a knowledge-based approach for interpreting genome-wide expression profiles. *Proceedings of the National Academy of Sciences of the United States of America* **102**, 15545–50 (2005).

[41] Beer, D. G. *et al.* Gene-expression profiles predict survival of patients with lung adenocarcinoma. *Nature medicine* **8**, 816–24 (2002).

[42] The Cancer Genome Atlas Research Network. Comprehensive genomic characterization defines human glioblastoma genes and core pathways. *Nature* **455**, 1061–8 (2008).

[43] The Cancer Genome Atlas Research Network. Comprehensive molecular portraits of human breast tumours. *Nature* **490**, 61–70 (2012).

[44] Rosner, B. Percentage Points for a Generalized ESD Many-Outlier Procedure. *Technometrics)* **25**, 165–172 (1983).

[45] Tebbutt, N., Pedersen, M. W. & Johns, T. G. Targeting the ERBB family in cancer: couples therapy. *Nature reviews. Cancer* **13**, 663–73 (2013).

[46] Minuti, G. & Landi, L. MET deregulation in breast cancer. *Annals of Translational Medicine* **3** (2015).

[47] Fernández-Medarde, A. & Santos, E. Ras in Cancer and Developmental Diseases. *Genes & Cancer* **2**, 344–358 (2011).

[48] Johnson, P. F. Molecular stop signs: regulation of cell-cycle arrest by C/EBP transcription factors. *Journal of Cell Science* **118**, 2545–2555 (2005).

[49] Mehner, C. *et al.* Tumor cell-produced matrix metalloproteinase 9 (MMP-9) drives malignant progression and metastasis of basal-like triple negative breast cancer. *Oncotarget* **5**, 2736–2749 (2014).

[50] Carvallo, L. *et al.* Non-canonical wnt signaling induces ubiquitination and degradation of syndecan4. *Journal of Biological Chemistry* **285**, 29546–29555 (2010).

[51] Montanaro, L., Treré, D. & Derenzini, M. Nucleolus, Ribosomes, and Cancer. *The American Journal of Pathology* **173**, 301–310 (2008).

[52] Pelletier, J., Thomas, G. & Volarević, S. Ribosome biogenesis in cancer: new players and therapeutic avenues. *Nature Reviews. Cancer* **18**, 51–63 (2018).

[53] Brennan, C. W. *et al.* The somatic genomic landscape of glioblastoma. *Cell* **155**, 462–77 (2013).

[54] Spalinger, M. R. *et al.* PTPN2 Regulates Inflammasome Activation and Controls Onset of Intestinal Inflammation and Colon Cancer. *Cell reports* **22**, 1835–1848 (2018).

[55] Visconti, R., Della Monica, R. & Grieco, D. Cell cycle checkpoint in cancer: a therapeutically targetable double-edged sword. *Journal of experimental & clinical cancer research : CR* **35**, 153 (2016).

[56] Perou, C. M. *et al.* Molecular portraits of human breast tumours. *Nature* **406**, 747–52 (2000).

Click here to access/download
Supplementary Material
cd_CAP_sup.pdf
