# Supplementary material for: Combinatorial Detection of Conserved Alteration Patterns for Identifying Cancer Subnetworks
Source: Gigascience. 2019 Apr 11;8(4):giz024. doi: 10.1093/gigascience/giz024 (PMC6458499; doi:10.1093/gigascience/giz024)
Supplement: response_to_reviewer_comments_original_submission.pdf [file giz024_response_to_reviewer_comments_original_submission.pdf]

Author's Response To Reviewer Comments

Close

Dear Dr. Zauner,

Following the reviewers' suggestions, we have made major structural improvements to the manuscript. In addition, we have addressed all of the problems and suggestions proposed by the reviewers in detail, including a comparison between our software tool and other existing methods, in a point-by-point 'Response to Reviewers' letter (uploaded as a Supplementary Material via the submission system). We agree to publish our revised manuscript as a "Technical Note" and we have created a "Technical Note" resubmission in the submission system. We have updated the manuscript according to the Technical Note format requirement from GigaScience. We have provided three sample datasets (which were used in the manuscript) in github repository https://github.com/ehodzic/cd-CAP and detailed instructions for users to run our program. We have also registered a new software application in the SciCrunch.org database with RRID SCR_016843 (see "Avilability of source code and requirements" in our revised manuscript).

Yours sincerely,
S. Cenk Sahinalp
Department of Computer Science,
Indiana University,
Bloomington, IN 47408, USA
cenksahi@indiana.edu

Close
