# Supplementary material for: Combinatorial Detection of Conserved Alteration Patterns for Identifying Cancer Subnetworks
Source: Gigascience. 2019 Apr 11;8(4):giz024. doi: 10.1093/gigascience/giz024 (PMC6458499; doi:10.1093/gigascience/giz024)
Supplement: response_to_reviewer_comments_revision_1.pdf [file giz024_response_to_reviewer_comments_revision_1.pdf]

Author's Response To Reviewer Comments

Close

Dear Dr. Zauner,

Following the reviewers' suggestions, we have made necessary improvements of the language and structure in our updated manuscript. We have submitted a point-by-point 'Response to Reviewers' letter as a supplementary material in the submission system.

Yours sincerely,
S. Cenk Sahinalp
Department of Computer Science,
Indiana University,
Bloomington, IN 47408, USA
cenksahi@indiana.edu

Close
