# Supplementary material for: Combinatorial Detection of Conserved Alteration Patterns for Identifying Cancer Subnetworks
Source: Gigascience. 2019 Apr 11;8(4):giz024. doi: 10.1093/gigascience/giz024 (PMC6458499; doi:10.1093/gigascience/giz024)
Supplement: response_to_reviewer_comments_revision_2.pdf [file giz024_response_to_reviewer_comments_revision_2.pdf]

Author's Response To Reviewer Comments

Close

Dear Dr Zauner,

Following the revision requirement in your email, we have included a reference to the GigaDB dataset and removed the highlighting red color in our manuscript. The LaTex source files for both the main text and supplementary material (along with the figures and bibliographies) are submited in attached files.

Thanks a lot

Yours sincerely,
S. Cenk Sahinalp
Department of Computer Science,
Indiana University,
Bloomington, IN 47408, USA
cenksahi@indiana.edu

Close
