# Supplementary material for: Combinatorial Detection of Conserved Alteration Patterns for Identifying Cancer Subnetworks
Source: Gigascience. 2019 Apr 11;8(4):giz024. doi: 10.1093/gigascience/giz024 (PMC6458499; doi:10.1093/gigascience/giz024)
Supplement: response_to_reviewer_comments_revision_3.pdf [file giz024_response_to_reviewer_comments_revision_3.pdf]

Author's Response To Reviewer Comments

Close

Dear Dr. Zauner,

Following the formatting requirement, we have moved the URL of our GigaDB reference which was appearing in the main body into the bibliography. We have resubmitted the corresponding tex and bibtex files.

Thanks,
S. Cenk Sahinalp
Department of Computer Science,
Indiana University,
Bloomington, IN 47408, USA
cenksahi@indiana.edu

Close
