# Supplementary material for: Combinatorial Detection of Conserved Alteration Patterns for Identifying Cancer Subnetworks
Source: Gigascience. 2019 Apr 11;8(4):giz024. doi: 10.1093/gigascience/giz024 (PMC6458499; doi:10.1093/gigascience/giz024)
Supplement: reviewer_1_report_original_submission -- Aaron McKenna — 8/27/2018 Reviewed [file giz024_reviewer_1_report_original_submission.pdf]

Reviewer Report

Title: Combinatorial Detection of Conserved Alteration Patterns for Identifying Cancer Subnetworks

Version: Original Submission **Date:** 8/27/2018

Reviewer name: Aaron McKenna

Reviewer Comments to Author:

Combinatorial Detection of Conserved Alteration Patterns for Identifying Cancer Subnetworks. Hodzic, Shrestha, and Zhu et al.
Hodzic, Shrestha, and Zhu et al. propose an amalgamation of existing computational approaches to discover important subnetworks that are mutated in tumors. Many aspects of the approach are novel and interesting, but the paper as a whole needs more structure and connection between sections, as well as general editing. For instance more explanation about when single-color and mixed colors are useful, how those networks compare in significance, etc. Clearing up these large issues would enable reviewers to make a more nuanced and fair review of the paper.
Major issues:
- This paper needs significant structural improvements. It's very hard to follow what's being done in what order, and how individual sections relate to each other.
- You could improve the clarity of the writing throughout the manuscript. For instance, it's challenging to understand the meaning of the second sentence of the motivation section, at least in my first pass reading it. The first paragraph of the 'our contributions section' is hard to understand as well. There are many other examples; a solid proofreading by the authors would be important before publication.
- Each node has to have the same alteration type? This would be my take-away from the first couple pages and figure 1. Then later in the paper you switch to multi-colored networks (for example figure 3). It's unclear in your 'colorful conserved networks' section if this needs to be mutually exclusive -- can you detect both at the same time?
- How do users select parameters like k and t?
- You talk about the co-amplification gain on chromosome arm 20q in COAD being an interesting finding, but doesn't your choice of a single alteration-type subnetwork bias you to find these large, common events?
- The 'options' for running cd-CAP are really hard to follow, with a mix of formatting styles (sometimes i or ii, sometimes a or b, which are all then compared at points?). This makes it really hard to understand the thought-process and evaluate the method.
- The authors should also compare this method to much earlier works that built statistical models of large contiguous losses of the genome such as GISTIC 2.0 (https://www.ncbi.nlm.nih.gov/pubmed/21527027)
- How do the subnetworks you identify compare, at the patient level, to other conventional classification approaches (such as CIMP+/- for COAD?)
- Can you compare your subnetwork against the survival curves of the most prognostic gene in 2G and J? How much of this predictive power is just picking the right single gene? The authors should also be

careful here, as many other factors (age, treatment, etc) could confound this, especially when 2G has such a strong skew in patient numbers and contains so many know cancer pathways.
Minor issues:
- The formatting right now looks like a CS paper, not a biology paper, but that's up to GigaScience.
- Pg 3 line 14. Complex processing procedures? What are you referring to?
- It would be worth adding something about tissue-specific function to the intro section, along the lines of Schneider et al.'s NRC paper: doi:10.1038/nrc.2017.5
- It would be good to give some indication of when your exhaustive search method breaks down (when how many genes are colored?)
- I appreciate the source code being available for review (I don't have the IBM CPLEX setup to test it, unfortunately).
- Are sample data sets available to run? This would help new users find and use the tool.

Level of Interest

Please indicate how interesting you found the manuscript: Choose an item.

Quality of Written English

Please indicate the quality of language in the manuscript: Choose an item.

Declaration of Competing Interests

Please complete a declaration of competing interests, considering the following questions:

- Have you in the past five years received reimbursements, fees, funding, or salary from an organisation that may in any way gain or lose financially from the publication of this manuscript, either now or in the future?
- Do you hold any stocks or shares in an organisation that may in any way gain or lose financially from the publication of this manuscript, either now or in the future?
- Do you hold or are you currently applying for any patents relating to the content of the manuscript?
- Have you received reimbursements, fees, funding, or salary from an organization that holds or has applied for patents relating to the content of the manuscript?
- Do you have any other financial competing interests?
- Do you have any non-financial competing interests in relation to this paper?

If you can answer no to all of the above, write 'I declare that I have no competing interests' below. If your reply is yes to any, please give details below.

I declare that I have no competing interests

I agree to the open peer review policy of the journal. I understand that my name will be included on my report to the authors and, if the manuscript is accepted for publication, my named report including any attachments I upload will be posted on the website along with the authors' responses. I agree for my report to be made available under an Open Access Creative Commons CC-BY license (http://creativecommons.org/licenses/by/4.0/). I understand that any comments which I do not wish to be included in my named report can be included as confidential comments to the editors, which will not be published.

I agree to the open peer review policy of the journal

To further support our reviewers, we have joined with Publons, where you can gain additional credit to further highlight your hard work (see: https://publons.com/journal/530/gigascience). On publication of this paper, your review will be automatically added to Publons, you can then choose whether or not to claim your Publons credit. I understand this statement.

Yes Choose an item.
