# Supplementary material for: Combinatorial Detection of Conserved Alteration Patterns for Identifying Cancer Subnetworks
Source: Gigascience. 2019 Apr 11;8(4):giz024. doi: 10.1093/gigascience/giz024 (PMC6458499; doi:10.1093/gigascience/giz024)
Supplement: reviewer_1_report_revision_1 -- Aaron McKenna — 12/15/2018 Reviewed [file giz024_reviewer_1_report_revision_1.pdf]

Reviewer Report

Title: Combinatorial Detection of Conserved Alteration Patterns for Identifying Cancer Subnetworks

Version: Revision 1 **Date:** 12/15/2018

Reviewer name: Aaron McKenna

Reviewer Comments to Author:

Overall this version of the paper is improved from the previous version. There are still some improvements to be made to the text for clarity and to reflect the underlying work correctly (at the discretion of GigaScience).
Issues:
The language is still very rough. I can point to some examples (the background of the abstract, the first sentence of the 'Maximal colorful subnetworks' section, etc) but it would be worth your effort to find someone unfamiliar with the manuscript to read it. An improved text would make the paper much more accessible to readers, and more likely to be cited.
Again, although this was put into the GigaScience Tex template, it still reads like a CS paper. The 'our contributions section' is an example. The material could be split between the introduction and methods sections, and this could cut down the length of the paper which would improve clarity.
There are a couple of places where you may want to tone down the language a bit. One example is saying the findings are 'clinically important' (page 2, column 2, like ~58) is a bit dangerous. You say the same for figure 2H in the text. You should be cautious (or avoid) making clinical claims. Again another read through the paper to ensure this and other minor claims are valid would be good.

Level of Interest

Please indicate how interesting you found the manuscript: Choose an item.

Quality of Written English

Please indicate the quality of language in the manuscript: Choose an item.

Declaration of Competing Interests

Please complete a declaration of competing interests, considering the following questions:

- Have you in the past five years received reimbursements, fees, funding, or salary from an organisation that may in any way gain or lose financially from the publication of this manuscript, either now or in the future?
- Do you hold any stocks or shares in an organisation that may in any way gain or lose financially from the publication of this manuscript, either now or in the future?

- Do you hold or are you currently applying for any patents relating to the content of the manuscript?
- Have you received reimbursements, fees, funding, or salary from an organization that holds or has applied for patents relating to the content of the manuscript?
- Do you have any other financial competing interests?
- Do you have any non-financial competing interests in relation to this paper?

If you can answer no to all of the above, write 'I declare that I have no competing interests' below. If your reply is yes to any, please give details below.

I declare that I have no competing interests

I agree to the open peer review policy of the journal. I understand that my name will be included on my report to the authors and, if the manuscript is accepted for publication, my named report including any attachments I upload will be posted on the website along with the authors' responses. I agree for my report to be made available under an Open Access Creative Commons CC-BY license (http://creativecommons.org/licenses/by/4.0/). I understand that any comments which I do not wish to be included in my named report can be included as confidential comments to the editors, which will not be published.

I agree to the open peer review policy of the journal

To further support our reviewers, we have joined with Publons, where you can gain additional credit to further highlight your hard work (see: https://publons.com/journal/530/gigascience). On publication of this paper, your review will be automatically added to Publons, you can then choose whether or not to claim your Publons credit. I understand this statement.

Yes Choose an item.
