# Supplementary material for: Combinatorial Detection of Conserved Alteration Patterns for Identifying Cancer Subnetworks
Source: Gigascience. 2019 Apr 11;8(4):giz024. doi: 10.1093/gigascience/giz024 (PMC6458499; doi:10.1093/gigascience/giz024)
Supplement: reviewer_2_report_original_submission -- Evan Paull — 10/2/2018 Reviewed [file giz024_reviewer_2_report_original_submission.pdf]

Reviewer Report

Title: Combinatorial Detection of Conserved Alteration Patterns for Identifying Cancer Subnetworks

Version: Original Submission **Date:** 10/2/2018

Reviewer name: Evan Paull

Reviewer Comments to Author:

The motivation and background discussion in this work are well motivated and the authors present a new method to find sets of genes that are recurrently altered in multiple samples, as well as connected within literature based pathway networks. Importantly, this method admits subnetworks that consist of multiple types of genomic or non-genomic alterations--i.e. copy number, SNVs, overexpression--which reflects true biology in that genes and protein products are known to be altered in a number of different ways.

The manuscript and methods are quite technical, and for clarity and readability I would suggest reordering the description of the problem statement. Specifically, the initial problem statement does not explain how the method is able to find 'colorful' subnetworks, but rather formulates the problem as one of single-color/alteration type networks. This is confusing as finding networks of multiple alteration types is the most useful application of this method, so I would suggest the authors start by clearly explaining that final goal at the beginning of the methods section. Details of the methodology could be explained after this initial problem statement, and all mathematical formulations should be clearly explained in the context of the specific alteration types they represent.

The second comment is simply a suggestion to include both a quantitative and qualitative comparison between this method and existing methods to find altered subnetworks. In particular, a visual comparison of partially overlapping subnetworks, so that the reader can clearly see the genes that are missed by each comparable method, as well as the region of overlap, would be highly informative and give insight into the strengths and weaknesses of this method. If completed, this would highly increase the visibility and impact of the final paper.

Level of Interest

Please indicate how interesting you found the manuscript: Choose an item.

Quality of Written English

Please indicate the quality of language in the manuscript: Choose an item.

Declaration of Competing Interests

Please complete a declaration of competing interests, considering the following questions:

- Have you in the past five years received reimbursements, fees, funding, or salary from an organisation that may in any way gain or lose financially from the publication of this manuscript, either now or in the future?
- Do you hold any stocks or shares in an organisation that may in any way gain or lose financially from the publication of this manuscript, either now or in the future?
- Do you hold or are you currently applying for any patents relating to the content of the manuscript?
- Have you received reimbursements, fees, funding, or salary from an organization that holds or has applied for patents relating to the content of the manuscript?
- Do you have any other financial competing interests?
- Do you have any non-financial competing interests in relation to this paper?

If you can answer no to all of the above, write 'I declare that I have no competing interests' below. If your reply is yes to any, please give details below.

I declare that I have no competing interests

I agree to the open peer review policy of the journal. I understand that my name will be included on my report to the authors and, if the manuscript is accepted for publication, my named report including any attachments I upload will be posted on the website along with the authors' responses. I agree for my report to be made available under an Open Access Creative Commons CC-BY license (http://creativecommons.org/licenses/by/4.0/). I understand that any comments which I do not wish to be included in my named report can be included as confidential comments to the editors, which will not be published.

I agree to the open peer review policy of the journal

To further support our reviewers, we have joined with Publons, where you can gain additional credit to further highlight your hard work (see: https://publons.com/journal/530/gigascience). On publication of this paper, your review will be automatically added to Publons, you can then choose whether or not to claim your Publons credit. I understand this statement.

Yes Choose an item.
